# Panchromatic Light Harvesting and Stabilizing Charge‐Separated States in Corrole–Phthalocyanine Conjugates through Coordinating a Subphthalocyanine

**DOI:** 10.1002/chem.202001442

**Published:** 2020-09-21

**Authors:** Beatrice Berionni Berna, Benedikt Platzer, Maximiliam Wolf, Giulia Lavarda, Sara Nardis, Pierluca Galloni, Tomás Torres, Dirk M. Guldi, Roberto Paolesse

**Affiliations:** ^1^ Department of Chemical Science and Technologies University of Rome Tor Vergata Via della Ricerca Scientifica 00133 Rome Italy; ^2^ Departamento de Química Orgánica Universidad Autónoma de Madrid, Campus de Cantoblanco C/ Francisco Tomás*y*Valiente 7 28049 Madrid Spain; ^3^ IMDEA—Nanociencia C/Faraday, 9. Campus de Cantoblanco 28049 Madrid Spain; ^4^ Institute for Advanced Research in Chemical Sciences (IAdChem) Universidad Autónoma de Madrid 28049 Madrid Spain; ^5^ Department of Chemistry and Pharmacy Interdisciplinary Center for Molecular Materials (ICMM) Friedrich-Alexander-Universitat Erlangen-Nürnberg Egerlandstr. 3 91058 Erlangen Germany

**Keywords:** electron transfer, energy transfer, organic photovoltaics, porphyrinoids, supramolecular chemistry

## Abstract

Owing to the electron‐donating and ‐accepting nature of corroles (Corr) and phthalocyanines (Pc), respectively, we designed and developed two novel covalently linked Corr‐Pc conjugates. The synthetic route allows the preparation of the target conjugates in satisfying yields. Comprehensive steady‐state absorption, fluorescence, and electrochemical assays enabled insights into energy and electron‐transfer processes upon photoexcitation. Coordinating a pyridine‐appended subphthalocyanine (SubPc) to the Pc of the conjugate sets up the ways and means to realize the first example of an array composed by three different porphyrinoids, which drives a cascade of energy and charge‐transfer processes. Importantly, the SubPc assists in stabilizing the charge‐separated state, that is, one‐electron oxidized Corr and the one electron‐reduced Pc, upon photoexcitation by means of a reductive charge transfer to the SubPc. To the best of our knowledge, this is the first case of an intramolecular oxidation of a Corr within electron‐donor–acceptor conjugates by means of just photoexcitation. Moreover, the combination of Corr, Pc, and SubPc guarantees panchromatic absorption across the visible range of the solar spectrum, with the SubPc covering the „green gap“ that usually affects porphyrinoids.

## Graphical abstract

**Cascades**: The preparation of two covalently linked corrole‐Zn phthalocyanine dyads is reported. The coordination of a pyridine‐appended perfluorinated subphthalocyanine to the zinc phthalocyanine leads to the formation of supramolecular triads. Corrole acts as electron donor upon photoexcitation, with subphthalocyanine as the final acceptor. These cascade‐like charge phenomena make these triads promising materials in molecular photovoltaics (see figure).

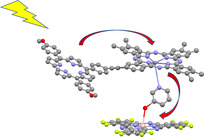

## Introduction

Organic photovoltaics has emerged as a major topic in contemporary research,^1^ using the photosynthetic systems found in nature as the main source of inspiration. To mimic the key steps of natural photosynthesis, significant efforts have been made in terms of the design and synthesis of a myriad of molecular architectures, in which the individual components have been adequately tailored and spatially arranged to imitate the antennas and/or reaction centers in plants.^2^ The predominant function of antennas is to secure a high rate and yield of charge transfer in the reaction center at low as well as high light intensities. Overall, the effectiveness of long‐range charge transfer relies on the suppression of charge recombination, that would interrupt the process and cause a waste of the absorbed energy.^3^ Despite the complexity of the photosynthetic conversion of light, designing, synthesizing, and probing multicomponent electron donor‐acceptor systems including covalent/non‐covalent multidiads (i.e. dyads, triads, tetrads, pentads, etc.) is at the forefront of investigations to optimize the rates and yields of energy and charge transfer.1c, 1i, 4

Specifically, an ideal (supra)molecular artificial solar energy conversion system should accomplish several requirements. First, antenna molecules should harvest light across most of the visible range of the solar spectrum with high extinction coefficients forming an „excited state species“. Second, the correspondingly formed excites states should be long‐lived and strong‐fluorescent. Third, the excited states should transfer its energy to either the energy or charge acceptors unidirectionally. Forth, the energetics of the resulting charge separated state should be high as well as close to that of the initially generated excited state, to minimize energy loss. And final, the rates of energy/charge transfer should outcompete those of the intrinsic deactivations of excited states. If in concert, that is, synchronized, all of the aforementioned should drive an efficient, unidirectional, and (semi)stable storage of converted light. Notably, a variety of multicomponent electron‐donor–acceptor systems have been subjected to photoinduced energy and charge‐transfer studies. Tetra‐ and tripyrrolic macrocycles play a key role in the composition of the aforementioned electron‐donor–acceptor systems: porphyrins (Pors),^5^ phthalocyanines (Pcs),^6^ corroles (Corrs),^7^ and subphthalocyanines (SubPcs),^8^ to just name a few.

Incentives for their use in these multicomponent arrays stem from their strong and tunable optical absorptions as well as their rich redox chemistry.^9^ Advanced photophysical investigations either in solution and/or in the solid state, revealed the occurrence, in the majority of the cases, of a charge transfer, that is, charge separation, charge transport, charge recombination, etc. upon photoexcitation.^10^ An important variable is the nature of the bridge both from an electronic (i.e. conjugated/non‐conjugated) and/or structural (i.e., rigid/flexible) point of view. This is meant to influence the charge‐transfer dynamics.^11^ To the best of our knowledge, no multicomponent systems based on corrole‐phthalocyanine (Corr‐Pc) arrays have ever been described to this date. Indeed, since Corr and Pc display complementary absorption with high absorption coefficients, they appear ideal light‐harvesting partners as they can cover together a large range of the solar spectrum (UV, visible and near IR). Moreover, the ease of corroles oxidation, if compared with the porphyrin analogues, makes them ideal candidates as electron donor in the multicomponent conjugate.^12^

In terms of electron acceptors, fullerenes have dominated the field of multicomponent electron‐donor–acceptor systems.^13^ Despite the fact that fullerenes promote efficient charge transfer, they feature a number of drawbacks. The limited possibility of modulating synthetic modifications and a low absorption intensity in the long wavelength visible region are just two of these drawbacks.

By going beyond the area of electron‐accepting fullerene, SubPcs have moved into the center of attention as a fullerene surrogate.^14^ Important is the fact that the electronic nature of SubPcs is modulated with ease by, for example, the introduction of electron‐withdrawing substituents at the periphery. Different synthetic routes have been developed to afford electron‐deficient SubPcs.^14^ SubPcs have intense absorptions in the 500 to 600 nm region and, in turn, help in filling the „green gap“ of porphyrinoid arrays in establishing panchromatic absorption across the visible range.

**Figure 1:**
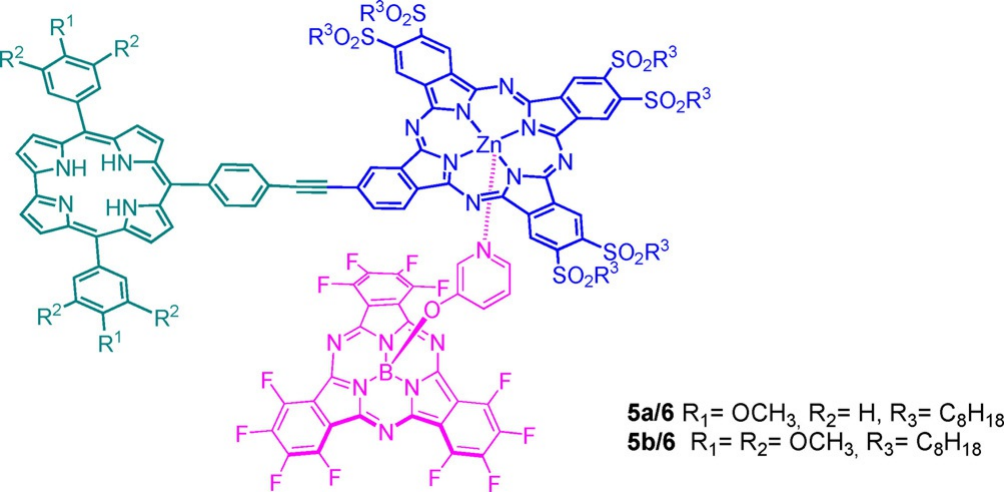
Chemical structure of the target molecules.

In the current work, we report the synthesis of two different Corr‐Pc electron‐donor–acceptor conjugates and their self‐assembly by means of metal coordination with a pyridine‐appended SubPc to afford multicomponent electron‐donor–acceptor arrays (Figure 1).

Our comprehensive experiments document an unprecedented electron transfer from the photoexcited electron‐rich Corr to the electron‐accepting Pc and followed by a charge shift to the SubPc. This sequence, constitutes a significant stabilization of the charge‐separated states relative to the simpler corrole‐phthalocyanine conjugate.

## Results and Discussion

### Synthesis

Considering the low symmetry of Corrs, functionalization at the corresponding β‐positions could result in a large number of different regioisomers. To exploit simple synthetic procedures, avoiding low yielding steps and tricky purifications we opted for a linkage at the Corr *meso*‐position. An additional notion stems from the evidence that *meso*‐carbons exhibit larger electronic densities than β‐carbons and, in turn, enabling stronger electronic couplings.^15^

**Scheme 1:**
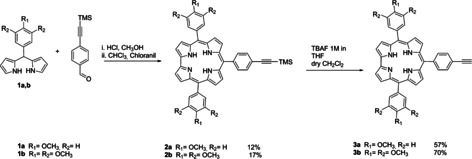
Synthesis of corroles **3 a,b**.

We prepared different (*meso*)Corr‐ZnPc electron‐donor–acceptor arrays, in which ZnPcs are linked to the 10‐*meso*‐ positions of Corrs via an ethynylphenyl spacer. To this end, we synthesized *trans*‐A_2_B Corrs bearing an ethynylphenyl moiety at their 10‐*meso*‐position, and an A_3_B phthalocyanine with a iodo functionality in one annulated benzene ring. In the case of Corrs, we reacted two different types of dipyrromethanes (DPMs), bearing different numbers of methoxy units at the phenyl group to enhance the electron‐donating character, with a 4‐trimethylsilylacetylene benzaldehyde to afford the desired *trans*‐A_2_B Corrs. A specific approach reported in the literature,^16^ particularly efficient for aldehydes bearing electron‐donating groups, was used. It involves the reaction of aldehydes with DPM in a water‐methanol mixture in the presence of HCl, using chloranil as the oxidant. Further treatment with tetra‐*n*‐butylammonium fluoride (TBAF) in dry CH_2_Cl_2_ removed the trimethylsilyl group affording corroles **3 a,b** in high yields (Scheme 1).

**3 a,b** were structurally established on the basis of their spectroscopic features, namely, UV‐vis‐absorption, ^1^H‐NMR, and mass analyses. Comparing the ^1^H‐NMR spectra of the corroles (Figure S**1**) major differences between the different substitution patterns are discernible. Features at *δ* 3.32–3.35 revealed the presence of an acetylenic proton. A different set of doublets between 7.4 and 8.4 ppm indicates the presence of a different number of protons on *meso*‐phenyl positions.

For **3 a**, we observed a 6H integration signal in the aliphatic zone, which underscores the presence of a methoxy moiety in *para*‐position of phenyl groups. Corrole **3 b** shows two signals with different integrations in the same zone, which is consistent with the presence of a trimethoxy functionality.

**Scheme 2:**
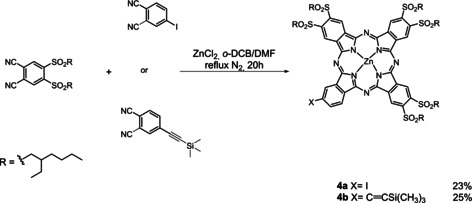
Synthesis of Pcs **4 a,b**.

Next, ZnPc **4 a,b** were obtained by a cyclotetramerization reaction of the desired 1,2‐dicyanobenzene derivative in a *o*‐dichlorobenzene/dimethylformamide (DMF) refluxing mixture and in the presence of Zn(OAc)_2_ as a template (Scheme 2).^17^

**Scheme 3:**
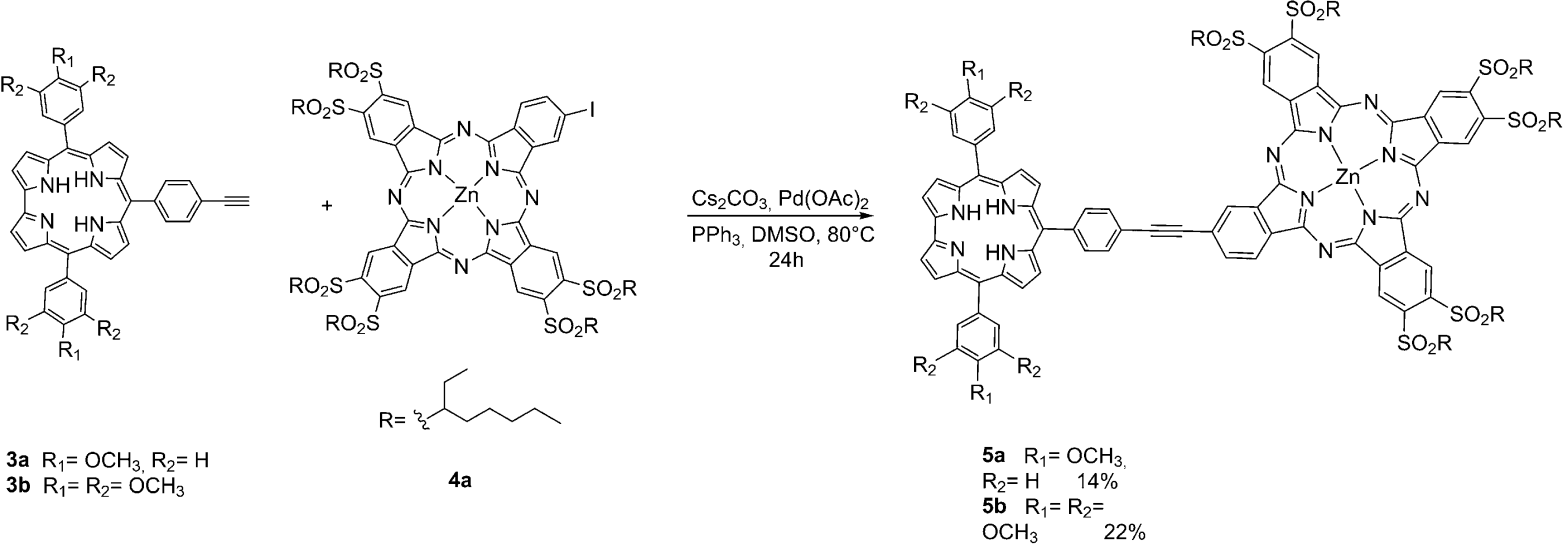
Synthesis of dyads **5 a**,**b**.

The presence of sulfonyl groups is expected to increase both the electron‐accepting character of the Pc and its solubility in organic solvents. A Sonogashira cross‐coupling reaction was identified as the means to form the link between Corr and ZnPc. We used the copper‐free methodology to prevent the metal insertion in the corrole ring during the reaction (Scheme 3).

**Scheme 4:**
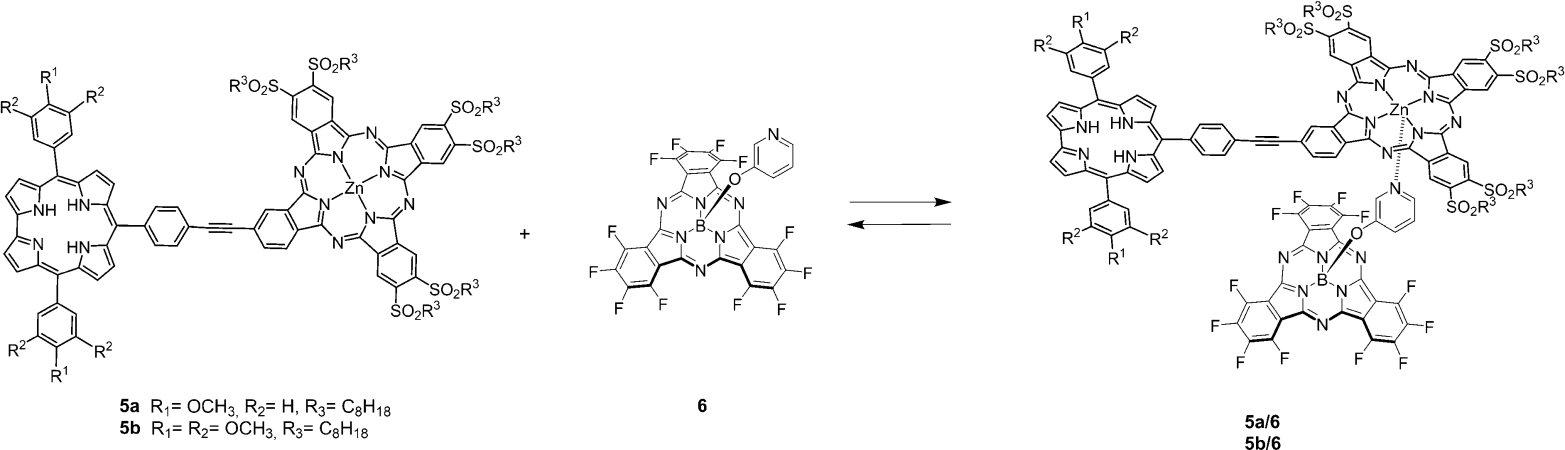
Metal‐ligand, axial coordination of perfluorinated SubPc **6** to dyads **5** **a** and **5** **b** leading to supramolecular complexes **5** **a/6** and **5** **b/6**.

The cross‐coupling reaction was carried out in dry dimethyl sulfoxide (DMSO) in the presence of Cs_2_CO_3_, Pd(OAc)_2_, and triphenylphosphine, with a Corr/ZnPc 1:1.2 molar ratio. The reaction mixture was stirred under argon at 80 °C for 20 hours. ^1^H NMR analysis of **5 a,b** showed spectra featuring distinct broadening of all the signals in the aromatic range, probably due to Pc aggregation. Nonetheless, MALDI‐TOF experiments corroborated the mass of **5 a,b**. Finally, **5 a,b** were connected to a perfluorinated subphthalocyanine **6** 18 by means of a supramolecular interaction (Scheme 4).

### Steady‐state absorption spectroscopy

In general, the absorption spectra of Corrs are dominated by intense Soret‐band absorptions, which maximize at around 425 nm, whereas ZnPcs show a broad and weak Soret‐band absorption in the range of 350 nm.^25^ Broad Q‐band absorption maxima are located in the case of a Corr reference between 510 and 650 nm. The Q‐band absorptions of ZnPcs are typically found in the range from 600 to 700 nm; the most prominent one is at around 675 nm as seen, for example, in the ZnT*t*BuPc reference. To this end, the absorption spectra of **2 a,b** feature an intense Soret‐band at 425 nm as well as Q‐bands between 520 and 660 nm. In order to deepen the photophysical properties of the single Pc unit, we decided to use **4 b** as a model ZnPc system. The presence of a trimethylsilylacetylene group, in lieu of the iodide moiety should provide to the Pc a chemical environment similar to that in dyads. In the case of **4 b** the broad Soret‐band and its four Q‐bands are found at 365 nm and between 610 and 710 nm, respectively.

**Figure 2:**
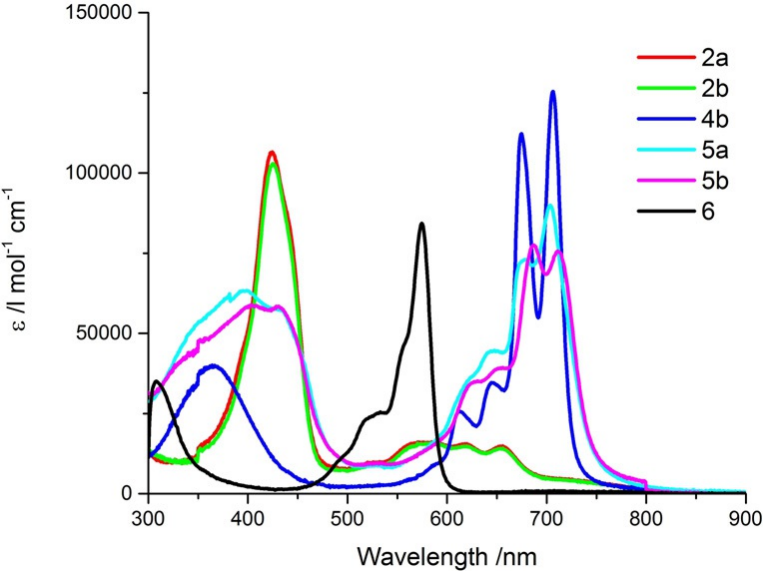
Steady‐state absorption spectra of Corrs **2 a**, **b**, ZnPc **4 b**, and Corr‐ZnPc conjugates **5 a,b** in toluene and SubPc **6** in anisole at room‐temperature.

Overall, **5 a,b** exhibit less intense and less defined Q‐band absorptions than **4 b**. Relative to **5 a**, a slight red‐shift that is discernable for **5 b**. The region below 500 nm gives rise to a broad band, which consists of the Soret‐band absorptions of Corrs and ZnPcs. Notably, we infer sizable ground‐state interactions as the absorption spectra of **5 a,b** cannot be reproduced from the sum of the spectra of **2 a,b** and **4 b**. To further substantiate the notion of ground state interactions, the molar extinction coefficients (Table 1) of **5 a,b** as well as ZnPc **4 b** and Corrs **2 a,b** were determined from the absorption spectra in toluene, anisole, and benzonitrile (Figures 2 and S11).

**Table 1:** Molar extinction coefficients *ϵ* (L mol^−1^ cm^−1^) and maxima (nm) of Soret‐ and Q‐band absorptions for Corrs **2 a**,**b**, ZnPc **4 b**, and Corr‐ZnPc **5 a**,**b** in different solvents at room‐temperature.

	**2 a**	**2 b**	**4 b**	**5 a**	**5 b**
toluene	1.07×10^5^ (424.5)	1.03×10^5^ (425)	1.12×10^5^ (675) 1.25×10^5^ (706.5)	7.3×10^4^ (679) 9.0×10^4^ (703.5)	7.8×10^4^ (687) 7.6×10^4^ (711)
anisole	8.4×10^4^ (424)	1.09×10^5^ (424.5)	1.02×10^5^ (677) 1.08×10^5^ (708.5)	7.5×10^4^ (681) 8.6×10^4^ (706)	6.6×10^4^ (687) 6.4×10^4^ (711)
benzonitrile	9.4×10^4^ (424.5)	8.6×10^4^ (425)	1.16×10^5^ (681) 1.25×10^5^ (714)	1.00×10^5^ (685) 1.07×10^5^ (713)	8.1×10^4^ (689) 7.8×10^4^ (716)

The corresponding absorption spectrum of 6 in anisole is also given in Figure 2. As a matter of fact, a mixture of the conjugate and the SubPc are seen throughout the visible range of the spectrum (Figure S12). As a complement, **5 a** and **6** were probed in ratios of up to 1:45 in anisole (Figure S13). The absorption spectra of **5 a** failed to exhibit any notable changes upon addition of 6. Likewise, the addition of **5 a** to **6** up to a ratio of 60:1 resulted in unaltered absorption spectra.

### Electrochemistry

In room temperature solution, Corr **3 a** showed two reversible oxidations at +0.44 and +0.79 V (Table 2). Similar is the oxidation of **3 b**, which displayed two reversible oxidations at +0.61 and +0.94 V, next to an additional irreversible oxidation at +0.44 V (Figure S8).^26^ In the case of ZnPc **4 b**, we observed two reversible reductions at −0.52 and −0.80 V as well as one reversible oxidation at +1.09 V (Figure S9). Compound **5 a,b** displayed two reversible reductions and one or two oxidation(s) (Figure S10). For **5 a**, the first and second reduction emerged at −0.47 and −0.77 V, respectively, while reversible oxidations are located at +0.61 and +1.20 V. For **5 b**, the reductions were found at −0.58 and −0.90 V, while the oxidation was seen at +0.61 V. Corrs 3a,b are far easier oxidized at +0.44 V than the electron‐deficient ZnPc **4 b** at +1.09 V,^17^ but harder to be reduced by nearly 0.5 V. The two reversible reductions of 5a,b are well in line with those of ZnPc **4 b**. A slightly more difficult reduction of **5 b** should be mentioned, that is, −0.58 versus −0.47 V. The low first oxidations at +0.61 V are comparable to those seen for Corrs **3 a,b**. In terms of charge‐separated states, that is, the one‐electron‐reduced ZnPc and the one‐electron‐oxidized Corr, their energies in dichloromethane were calculated to be +1.08 and +1.19 V for **5 a** and **5 b**, respectively.

**Table 2:** Redox potentials versus standard calomel electrode of **3 a**, **3 b**, **4 b**, **5 a** and **5 b** in CH_2_Cl_2_

	*E*_red(2)_ [V]	*E*_red(1)_ [V]	*E*_ox 1_ [V]	*E*_ox 2_ [V]	*E*_ox 3_ [V]
**3 a**	–	−1.01^[a]^	0.44	0.79	–
**3 b**	–	–	0.44^[a]^	0.61	0.94
**4 b**	−0.80	−0.52	1.09	–	–
**5 a**	−0.77	−0.47	0.61	1.20	–
**5 b**	−0.90	−0.58	0.61	–	–

[a] Irreversible.

### Steady‐state fluorescence spectroscopy

**Figure 3:**
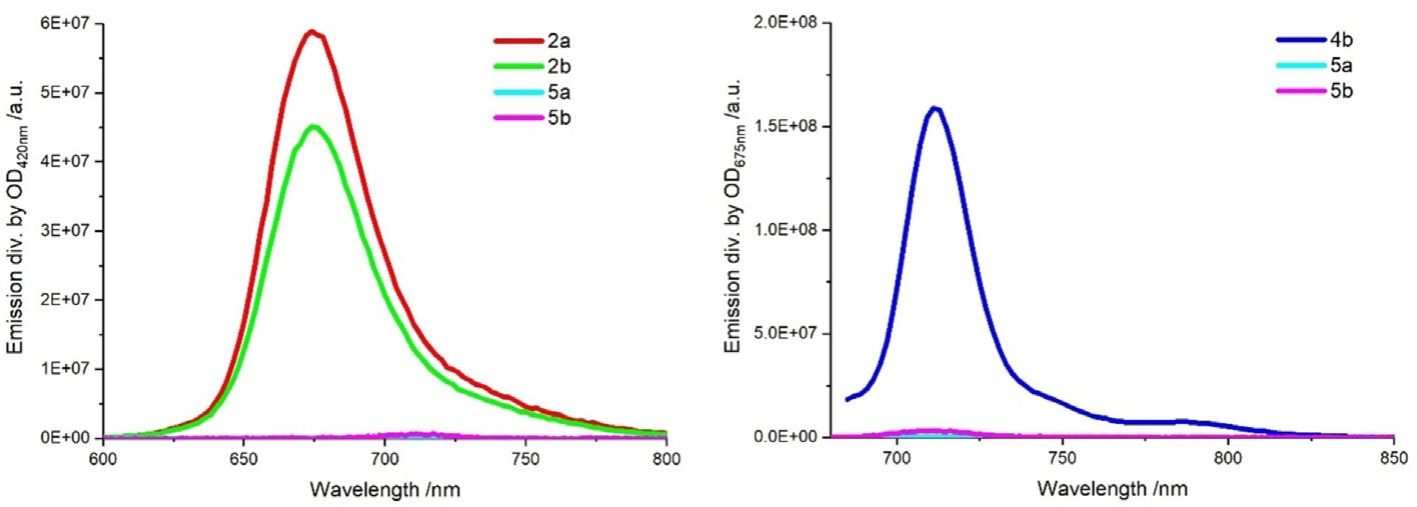
Steady‐state fluorescence spectra of (left) **2 a,b** and **5 a,b** upon excitation at 420 nm and (right) **4 b** and **5 a,b** upon excitation at 675 nm with given fluorescence intensity divided by the respective OD at the excitation in toluene and acquired at room‐temperature.

Fluorescence spectra were acquired in various solvents, namely toluene (Figure 3), anisole, and benzonitrile. The fluorescence maxima as they were recorded upon excitation in the regions of the Soret‐ or Q‐band absorptions at 420 nm and 675 nm, respectively, are given in Table 3.

**Table 3:** Fluorescence maxima for **2 a,b**, **4 b** and **5 a,b** in toluene, anisole and benzonitrile upon excitation at 420 or 675 nm (corresponding energy in eV in brackets).

	**2 a**	**2 b**	**4 b**	**5 a**	**5 b**
toluene	674 (1.84)	674 (1.84)	711 (1.74)	711 (1.74)	714 (1.74)
anisole	677 (1.83)	677 (1.83)	716 (1.73)	712 (1.74)	715 (1.73)
benzonitrile	680 (1.82)	680 (1.82)	722 (1.72)	718 (1.73)	720 (1.72)

For **2 a,b**, the fluorescence spectra upon 420 nm photoexcitation feature a single maximum at 674 nm in, for example, toluene, which is subject to an appreciable red‐shift in more polar solvents. In the case of **4 b** the spectra consist of a maximum upon 675 nm photoexcitation, which evolves at around 711 nm in non‐polar solvents and at around 722 in polar solvents. For **5 a,b**, the results show fluorescence solely from ZnPc in the range of 700 nm and beyond regardless of the excitation wavelength. This prompts to efficient electron transfer from the energetically higher lying Corr singlet excited state to that of the lower lying ZnPc. Independent evidence for this interpretation comes from 3D plots taken in toluene (Figure S15). Red‐shifts of several nanometers are also noted with increasing solvent polarity. Quantum yields were determined by means of a comparison to a TPP reference (420 nm), a ZnT*t*BuPc reference (675 nm), and a perfluorinated SubPc, whose central boron features a phenoxy group with corresponding values of 0.11, 0.3 and 0.17, respectively.^19^ Quantum yields of far less than 1 % point towards fast alternative deactivation pathways of the excited states of **5 a,b** when compared to the rather high yields of **2 a,b** and **4 b** (Table 4). The SubPc **6** also shows strong emission with an emission maximum at 580 nm (Figure S16) and a calculated quantum yield of 0.19. First insights into excited state interactions between **5 a** and **6** came from fluorescence spectroscopy. Interestingly, the fluorescence of **6** decreases upon addition of **5 a** in anisole (Figure S17) from which we conclude the successful coordination, on one hand, and excited state interactions between Corr‐/ZnPc and SubPc, on the other hand. A monoexponential fit was used to determine the corresponding association constant of 3.4×10^4^ m ^−1^.

**Table 4:** Fluorescence quantum yields for **2 a,b**, **4 b** and dyads **5 a,b** in toluene, anisole, and benzonitrile upon excitation at either 420 or 675 nm.

	**2 a**	**2 b**	**4 b**	**5 a**		**5 b**	
*λ*_Exc_ [nm]	420	420	675	420	675	420	675
toluene	0.13	0.11	0.19	–	<0.001	0.002	0.007
anisole	0.15	0.18	0.23	<0.001	0.003	<0.001	0.005
benzonitrile	0.12	0.13	0.21

### Spectroelectrochemistry

**Figure 4:**
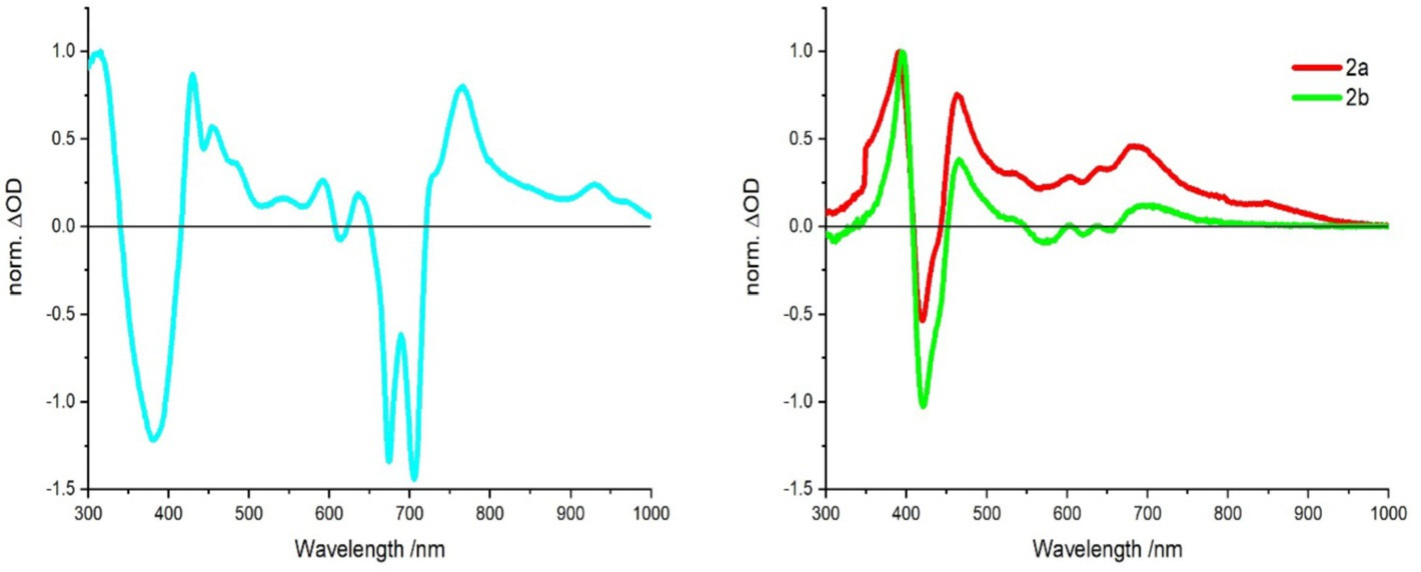
Differential absorption spectra obtained upon electrochemical reduction of the corrole‐phthalocyanine **5 a** at an applied bias of −0.9 V in ODCB (including 0.1 m *n*Bu_4_NClO_4_ as electrolyte; left) and electrochemical oxidation of the free‐base corroles **2 a** and **2 b** at an applied bias of +1.1 V in DCM (including 0.1 m *n*Bu_4_NPF_6_ as electrolyte; right) after solvent‐saturation with Argon at room‐temperature and silver‐wire as pseudo‐reference electrode.

Spectroelectrochemical measurements were conducted to assist in the analysis of the transient absorption measurements—vide infra. To this end, the differential absorption spectra of the one‐electron‐reduced form of ZnPc (**5 a**) and the one‐electron‐oxidized form of Corr (**2 a** and **2 b**) were monitored spectroscopically upon applying either a reductive or an oxidative bias, respectively (Figure 4). In the case of **5 a**, the one‐electron‐reduced form of ZnPc reveals maxima at 311, 430, 456, 542, 591, 636, 765, and 931 nm as well as minima at 380, 674, and 706 nm.

Notably, the features in the visible region closely resemble those recorded for the one‐electron‐reduced form of a ZnT*t*BuPc reference.13c The one‐electron‐oxidized form of the two Corrs, that is, **2 a** and **2 b**, are quite similar with maxima at around 390, 465, 603, 638, and 690 nm and minima at 421, 570, 618, and 653 nm. Differential spectra upon reduction of the perfluorinated SubPc 6 (Figure S18) show maxima at around 460 and 660 nm as well as a minimum at 573 nm.

### Femtosecond transient absorption spectroscopy

#### Femtosecond transient absorption spectroscopy of the covalent dyad

Insights into excited‐state dynamics were gathered via transient absorption spectroscopy in anisole, while GloTarAn global and target analyses^20^ were performed with the data. Starting with the references, **4 b** initially displays multiple features upon photoexcitation into the Q‐band absorption at 676 nm (Figure S**19**). In anisole, we observed singlet excited state (*S_1_) characteristics, including maxima at 518, 630, 840 and 1230 nm, a shoulder at 595 nm, and minima at 679 and 712 nm. GloTarAn global analysis,^20^ based on a two species model, reveals that all of these features give rise to a 2.7 ns lifetime. They transform monoexponentially into the triplet excited state (*T_1_) with characteristic maxima, which are found at 420–600, 779, and 1546 nm.

Next, **2 a** was probed upon 430 nm photoexcitation, that is, into the Soret‐band absorption (Figure S**20**) in anisole. Three species were needed for the GloTarAn global analysis^20^ in the visible range, while two were sufficient for the NIR range. Initially, population of the second singlet excited state (*S_2_) with maxima between 470 and 580 nm as well as at 780 and 993 nm is followed by the first singlet excited state (*S_1_) with similar maxima at 470, 545 and 800 nm. The third species, which is assigned to the triplet excited state (*T_1_), also shows a maximum at 470 nm, which is, however, accompanied by a shoulder at 530 nm. Notable is the fact that the amplitude of (*T_1_) is low. This causes a much poorer signal‐to‐noise ratio in the corresponding Evolution Associated Spectra (EAS). The obtained lifetimes for *S_2_, *S_1_, and *T_1_ are 4 ps, 80 ps, and several (>5) ns, respectively.

**Figure 5:**
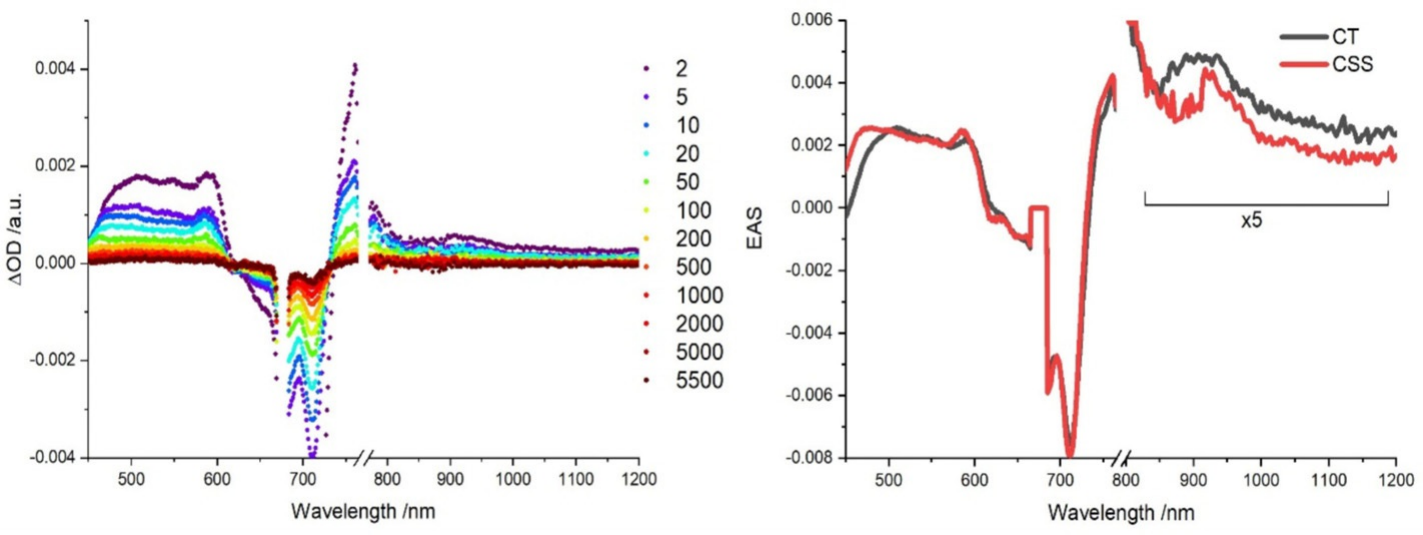
(left) Differential absorption spectra with delays between 2 and 5500 ps and (right) evolution associated spectra of transient species of **5 a** obtained upon femtosecond flash‐photolysis (excitation at 676 nm) in de‐aerated anisole at room‐temperature.

Differential absorption spectra upon 676 nm photoexcitation into the ZnPc Q‐band absorption of dyad **5 a** display distinct maxima at 505, 590, 760, and a broad maximum at 900 nm (Figure S21).^27^ These features, but especially those at 590 and 760 nm, are in good agreement with the absorption of the one‐electron‐reduced form of ZnPc. Deconvolution via a two species consecutive kinetic model afforded states with lifetimes of ca. 1 and 70 ps. Both species show the absorption of the aforementioned characteristics and should, in turn, be a Corr^δ+^‐ZnPc^δ−^ charge‐transfer (CT) state, if not a Corr^⋅+^‐ZnPc^⋅−^ charge‐separated (CS) state. The second species furthermore features an even broader signal, which reaches all the way to 470 nm, as well as two remaining minor maxima at 930 and 960 nm. These transients prompt to the one‐electron‐oxidized and reduced form of Corr and ZnPc, respectively. In summary, **5 a** is photoexcited into a CT state followed by a CS state with respective lifetimes of around 1 and 70 ps. We cannot rule out the initial occupation of a singlet excited (*S_1_) state, but it decays faster than the instrumental resolution (Figure 5).

**Figure 6:**
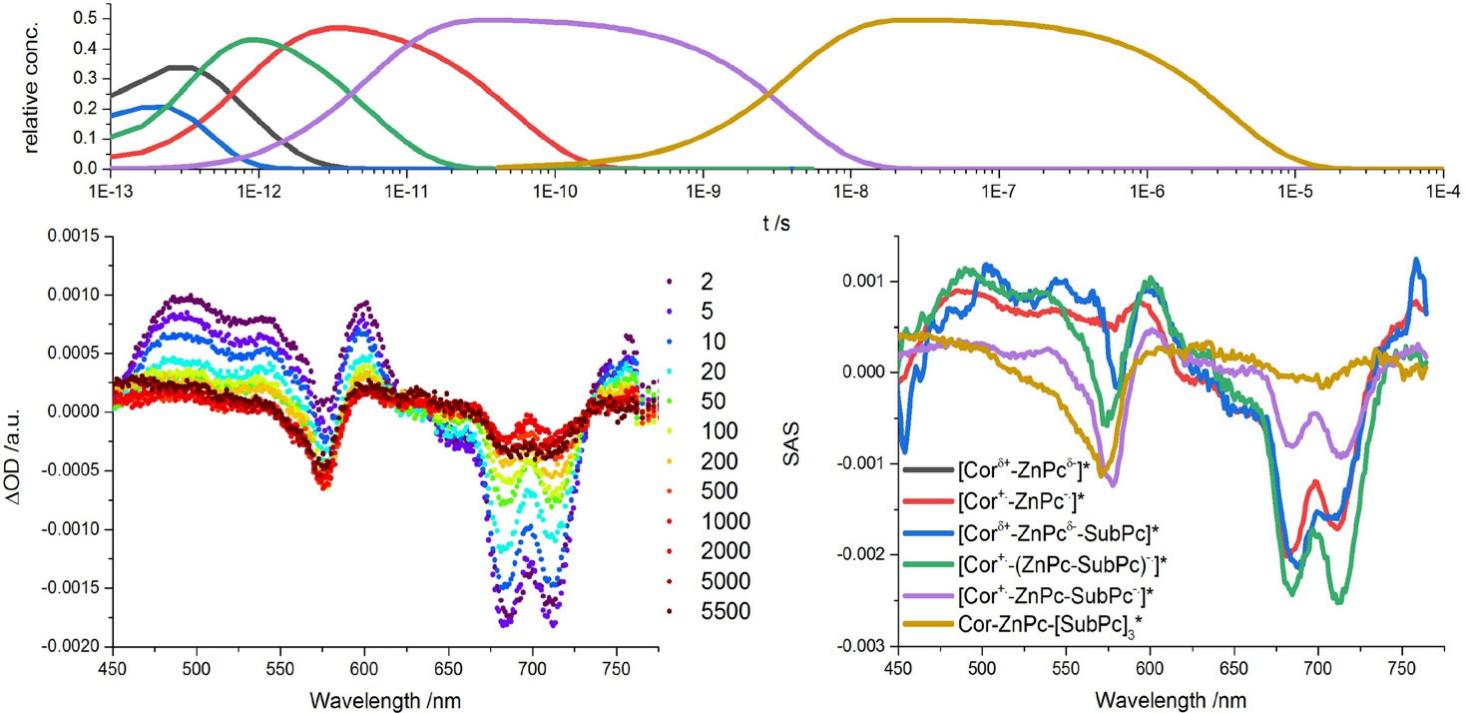
(left) Differential absorption spectra with delays between 2 and 5500 ps, (right) species associated spectra of transient species of a 1:20 mixture of **5 a** and **6** obtained upon femtosecond flash‐photolysis and (top) relative concentration profiles obtained upon femtosecond and nanosecond flash‐photolysis (excitation at 430 nm) in de‐aerated anisole at room‐temperature.

When turning to Corr photoexcitation in **5 a** at 430 nm (Figure 6) fingerprints, which are associated with the one‐electron‐reduced form of ZnPc occur at 590 and 760 nm. EAS of the first and also the second species obtained from GloTarAn global analysis show these spectroscopic markers, which leads us the assign them as a CT and a CS state, respectively. The respective lifetimes are about 1 and 55 ps. We must assume that prior to the CT and CS states, a highly energetic singlet excited (*S_1_) state has been populated upon photoexcitation and converted into the CT state faster than our instrumental response.

Similar results were gathered for **5 b**. In particular, GloTarAn global analysis upon either 430 nm photoexcitation of Corr or 676 nm photoexcitation of ZnPc yielded the same CT state and CS states as first and second species, respectively (Figures S**19** and **S20**). CT and CS lifetimes are 7 and 120 ps, respectively, upon Corr photoexcitation and 5 and 90 ps, respectively, upon ZnPc photoexcitation. The longer CT and CS lifetimes are rationalized in **5 b** by their slightly higher energies compared to **5 a**.

#### Femtosecond transient absorption spectroscopy of the supramolecular triad

Finally, **5 a** and **6** were probed in a 1:20 ratio to afford **5 a,b** vs. **5 a,b**/**6** in equal yields upon 430 nm photoexcitation of Corr in anisole (Figure 6). At first glance, the fingerprint absorption of the one‐electron‐reduced form of SubPc is discernible throughout the measurements. A transfer of charges is the only feasible pathway following photoexcitation of Corr. GloTarAn target analysis was conducted and resulted into six species assuming the coexistence of **5 a** with and without **6**. Consequently, the first and second species are the CT and short‐distance CS states, respectively, as seen for **5 a** and **5 b**. They ultimately undergo charge recombination to afford the ground state within the sub‐nanosecond range. Next to the aforementioned is the third species, which relates to the same CT state, but with **6** being coordinated to **5 a**, which is populated directly upon excitation.

This CT state transforms into the short‐distance CS state with 0.2 ps.^28^ Interestingly, this species features already the spectroscopic fingerprint of the one‐electron‐reduced form of SubPc in the form of the SubPc‐centered bleaching at 575 nm. As such, we infer a delocalization of negative charges between the electron‐accepting ZnPc and SubPc. Much stronger is the SubPc‐centered bleaching relative to that of ZnPc in the next species, which emerges with 6 ps. Here, an almost full localization of the negative charges on the SubPc is realized and relates to the long‐distance CS state. Charge recombination within this long‐distance CS state ultimately occurs with 4.0 ns. The product of charge recombination is, however, the SubPc triplet excited state rather than the ground state. The latter is formed with tens of microseconds. Complementary, nanosecond transient absorption measurements (Figure S24) also reveal the formation of the long‐distance CS state with little or no ZnPc‐centered bleaching and the SubPc triplet excited state.

**Figure 7:**
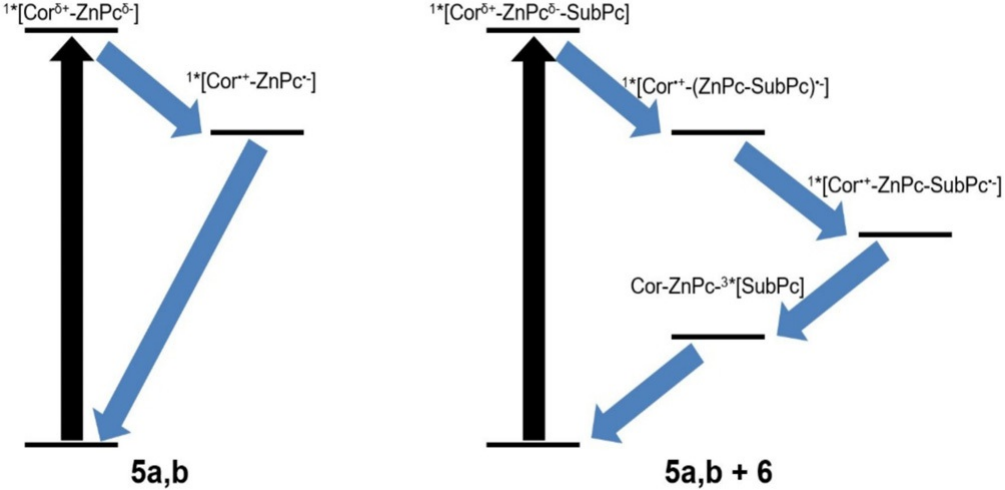
Kinetic model employed for target analysis of transient absorption data of 1:20 mixtures of **5 a**,**b** and SubPc **6** based on experiments in de‐aerated anisole.

The evolution of all the species over time is summarized in Figure 7. A quantitative similar picture was gathered upon 430 nm excitation of a 1:20 mixture of **5 b** and **6** in the target analyses of the femtosecond (Figure S24) and nanosecond transient absorption data (Figure S26). Charge separation occurs on the sub‐picosecond time scale with 0.2 ps, while the shift of charges from ZnPc to SubPc is seen to take place with 3 ps. Recombination of charges in the long‐distance CS state finally takes place on the scale of nanoseconds, that is,6.4 ns. In short, effective charge separation as well as charge shift in the short‐distance CS state to ultimately form the long‐distance CS states for **5 a**/**6** and **5 b**/**6** upon Corr excitation, which is two orders of magnitude longer lived than the CS states for **5 a** and **5 b** (Figure 7).

## Conclusion

Three different porphyrinoids, that is, corroles (Corr), phthalocyanines (Pc), and subphthalocyanine (SubPc) have been integrated into a novel conjugate. A synthetic route has been defined to covalently link Corr and Pc, while a pyridine appended SubPc has been coordinated to the Pc. Corr acts as electron donor after photoexcitation, while Pc and SubPc function as primary and secondary electron acceptor, respectively. Importantly, the presence of Pc enables an effective charge separation between Corr and Pc, on one hand, and charge shift to SubPc, on the other hand. The consequently formed Corr^⋅+^‐ZnPc‐SubPc^⋅−^ CS state is two orders of magnitude longer lived than the Corr^⋅+^‐ZnPc^⋅−^‐SubPc CS state. Our study demonstrates that the careful choice of a series of porphyrinoids with an appropriate energy level alignment enables the formation of long‐lived CS states once photoexcited, without, however, the need of a fullerene as final electron acceptors. The sum of these cascade‐like phenomena render the Corr‐ZnPc‐SubPc ensembles a model photosynthetic reaction center and enables the exploitation of these promising materials in molecular photovoltaics.

## Experimental Section

### Material and general methods

Chemical reagents and solvents (Sigma–Aldrich, Alfa Aesar, Fluka Chemie and Carlo Erba Reagenti) were of the highest grade available and were used without further purification. Additionally, some solvents were further dried by distillation with LiAlH_4_ (THF, toluene), or with previously activated molecular sieves (3 or 4 Å), or with a solvent purifying system by Innovative Technology Inc. MD‐4‐PS. Air‐ and moisture sensitive experiments were carried out using standard Schlenk line techniques. Chromatography: Thin layer chromatography (TLC) analyses were performed on aluminum sheets coated with silica gel 60 F254 or neutral alumina 60 F254 (Merck). TLC analyses were carried out with an UV lamp of 254 and 365 nm. Column chromatography was carried out using silica gel Merck‐60 (230–400 mesh, 60 Å), Sigma–Aldrich (70–230 mesh, 60 Å) and and neutral alumina (Merk, Brockmann Grade III) as the solid support. For the dyads, the final compounds were purified by size‐exclusion chromatography on BioBeads SX1 (BioRad). Eluents and relative proportions are indicated for each particular case. Nuclear magnetic resonance (NMR): ^1^H and ^13^C NMR spectra were measured on a a Bruker AC‐300 (300 MHz) or a Bruker Avance III 400 BBFO spectrometer, locked on deuterated solvents. Chemical shifts are given in ppm relative to residual solvents using literature reference of δ ppm values.^21^ Mass‐spectrometry (MS): Matrix‐assisted laser desorption/ionization time of flight (MALDI‐TOF) was recorded with a Bruker Ultrareflex III spectrometer that was equipped with a nitrogen laser operating at 337 nm and recorded in the positive‐polarity mode. The different matrixes employed are indicated for each spectrum. Mass spectrometry data are expressed in *m*/*z* units. Steady state absorption spectroscopy was carried out with a Lambda 2 UV/Vis/NIR double beam spectrometer from PERKINELMER (190 to 1100 nm) and a Cary 5000 UV/Vis/NIR double beam spectrophotometer from VARIAN (175 to 3300 nm). The data was recorded with the software UV WinLab using a slit width of 2 nm and a scan rate of 480 nm min^−1^ and 600 nm min^−1^, respectively. All measurements were performed without deaerating in a 10×10 mm Quartz cuvette (QS) with the respective solvent as reference. Steady state fluorescence measurements were performed with a FluoroMax®‐3 fluorometer from HORIBA JOBIN YVON in a 10×10 mm Quartz cuvette (QS) without deaerating. All spectra were corrected for the instrument response. Femtosecond transient absorption spectroscopy: All spectra were obtained with a Ti:sapphire laser system CPA‐2101 (Clark‐MXR, Inc.) in combination with a Helios TAPPS‐transient absorption pump probe spectroscopy detection unit from Ultrafast Inc. The initial laser output wavelength was 633 nm with a pulse width of 150 fs and 1 kHz repetition. The excitation wavelength was generated using NOPA noncollinear optical parametric amplifier. Transient absorption spectra were measured in THF in a 0.6 mL cuvette with a 0.2 cm path length. Finally, spectra were acquired with a HELIOS (Ultrafast Systems) transient absorption spectrometer. Electrochemistry: cyclic voltammetry (CV) experiments were performed with a Palmsens potentiostat, using a standard calomel electrode (SCE) as the reference electrode, a platinum wire as the auxiliary electrode and a platinum disk (1 mm diameter) as working electrode. The experiments were performed in anhydrous CH_2_Cl_2_ using tetrabutylammonium perchlorate (TBAP, Aldrich, electrochemical grade) as supporting electrolyte at 100 mV s^−1^ scan rate. Prior to each voltammetric measurement, the cell was degassed by bubbling with nitrogen for about 20 min. Electrochemical measurements were performed using a concentration of approximately 1×10^−3^ m for the compound in question. Compensation for internal resistance was not applied. Spectroelectrochemistry: spectroelectrochemistry measurements were done in a home‐made three neck glass cell. The used three‐electrode setup was comprised of a platinum mesh as working electrode, a Ag‐wire as pseudo reference electrode, and a platinum wire as counter electrode. All measurements were performed either in *o*‐DCB with 0.1 m *n*Bu_4_NClO_4_ as supporting electrolyte or DCM with *n*Bu_4_NPF_6_ at concentration 0.1 M. All measurements were performed at room temperature.

### Synthetic procedures

Dipyrromethanes **1 a**,**b**,^22^ corroles **2 a**,**b**,^16^ phthalonitriles,^23^ Pcs **4 a**,**b**,^17^ and SubPc **6** 18b were prepared following literature procedures.

### General procedure for the syntheses of *trans*‐A_2_B corroles

Samples of 4‐[(trimethylsilyl)ethynyl]benzaldehyde (0.9 mmol) and dipyrromethanes (1.8 mmol) were dissolved in methanol (90 mL). Subsequently, a 90 mL solution of HCl (36 %) in H_2_O was added, and the reaction was stirred at r.t. for 90 min.

The progress of the reaction was monitored by periodic removal of aliquots from the reaction mixture followed by TLC examination. The crude product was diluted with CHCl_3_ (25 mL) and washed twice with H_2_O. The mixture was dried over Na_2_SO_4_ and evaporated (in order to remove the residual CH_3_OH). Then CHCl_3_ (450 mL) was added, followed by the addition of 2.7 mmol of chloranil. The reaction was stirred at room temperature for 2 hours. The crude product was purified on a silica gel column; different eluent mixtures and characterization data of all the newly prepared compounds are given below.

**5,15‐Bis(4‐methoxyphenyl)‐10‐[(4‐trimethylsilyl)ethynyl]phenylcorrole (2 a)**: Chromatography using CH_2_Cl_2_ as eluent. Yield: 12 %, green solid, mp >300 °C; ^1^H NMR (300 MHz, CDCl_3_, *δ* ppm): 0.38 (s, 9 H, SiCH_3_), 4.08 (s, 6 H, OCH_3_), 7.36 (br d, 4 H, *meso*‐Ar), 7.86 (br d, 2 H, *meso*‐Ar), 8.12 (br d, 2 H, *meso*‐Ar), 8.28 (br d, 4 H, *meso*‐Ar), 8.48 (br d, 2 H, β‐pyrr), 8.56 (br d, 2 H, β‐pyrr), 8.86 (br d, 2 H, β‐pyrr), 8.93 (br d, 2 H, β‐pyrr); Anal. Calcd for C_44_H_38_N_4_O_2_Si: C, 77.39; H, 5.61; N, 8.20; found: C, 77.32; H, 6.68; N, 8.21; MS (FAB, *m*/*z*): 684 (M^+^); UV‐vis (CH_2_Cl_2_): *λ*_max_ nm (log *ϵ*): 420 (5.22), 438 (sh), 583 (4.43), 620 (4.43), 654 (4.34).

**5,15‐Bis(3,4,5‐trimethoxyphenyl)‐10‐[(4‐trimethylsilyl)ethynyl]phenylcorrole (2 b)**: Chromatography using CHCl_3_ as eluent. Yield: 17 %, green solid, mp >300 °C. ^1^H NMR (300 MHz, CDCl_3_, *δ* ppm): 0.43 (s, 9 H, SiCH_3_), 4.14 (s, 12 H, OCH_3_), 4.20 (s, 6 H, OCH_3_), 7.66 (s, 4 H, *meso*‐Ar), 7.92 (d, 2 H, *J*=6 Hz, *meso*‐Ar), 8.16 (d, 2 H, *J*=6 Hz, *meso*‐Ar), 8.57 (d, 2 H, *J*=3 Hz β‐pyrr), 8.72 (d, 2 H, *J*=3 Hz β‐pyrr), 9.02 (br m, 4 H, β‐pyrr); Anal. Calcd for C_48_H_46_N_4_O_6_Si: C, 71.80; H, 5.77; N, 6.98; found: C, 71.79; H, 5.83; N, 6.94; MS (MALDI‐TOF, matrix DCTB *m*/*z*): 802.4 (M^+^); UV‐vis (CH_2_Cl_2_): *λ*_max_ nm (log *ϵ*): 425 (4.91), 580 (3.96), 623 (4.05), 655 (4.21).

**General procedures for the removal of trimethylsilyl group**: Corrole (0.14 mmol) was added to a 14 mL solution of dry CH_2_Cl_2_, then TBAF (123 μL of a 1 m solution in THF) was added. The mixture was allowed to stir for 2 h at rt; until all substrate was consumed. When no more starting material was detected on TLC analysis, the solvent was removed under reduced pressure. The crude was taken up with CHCl_3_ and washed twice with water; then it was dried on Na_2_SO_4_. After filtration of the drying agent, the solvent was evaporated. Chromatographic purification of the reaction crude was performed on a silica gel column, eluting with CHCl_3_. Characterization data of all the newly prepared compounds are given below.

**5,15‐Bis(4‐methoxyphenyl)‐10‐(4‐ethynylphenyl)corrole (3 a)**: Yield: 57 %, green solid, mp >300 °C. ^1^H NMR (300 MHz, CDCl_3_, *δ* ppm): 3.32 (s, 1 H, CH), 4.14 (s, 6 H, OCH_3_), 7.42 (d, 4 H, *J*=6 Hz, *meso*‐Ar), 7.94 (d, 2 H, *J*=6 Hz, *meso*‐Ar), 8.20 (d, 2 H, *J*=6 Hz, *meso*‐Ar), 8.34 (d, 4 H, *J*=6 Hz, *meso*‐Ar), 8.56 (d, 2 H, *J*=3 Hz, β‐pyrr), 8.62 (d, 2 H, *J*=3 Hz, β‐pyrr), 8.92 (d, 2 H, *J*=3 Hz, β‐pyrr), 8.98 (d, 2 H, *J*=3 Hz, β‐pyrr). Anal. Calcd for C_41_H_30_N_4_O_2_: C, 80.63; H, 4.95; N, 9.17; found: C, 80.70; H, 4.93; N, 9.23; MS (MALDI‐TOF, matrix DCTB *m*/*z*): 610.3 (M^+^); UV‐vis (CH_2_Cl_2_): *λ*_max_ nm (log *ϵ*): 421 (5.16), 438 (sh), 582 (4.37), 622 (4.39), 655 (4.36).

**5,15‐Bis(3,4,5‐trimethoxyphenyl)‐10‐(4‐ethynylphenyl)corrole (3 b)**: Yield: 70 %, green solid, mp >300 °C. ^1^H NMR (300 MHz, CDCl_3_, *δ* ppm): 3.35 (s, 1 H, CH), 4.14 (s, 12 H, OCH_3_), 4.21 (s, 6 H, OCH_3_), 7.67 (s, 4 H, *meso*‐Ar), 7.95 (d, 2 H, *J*=6 Hz, *meso*‐Ar), 8.21 (d, 2 H, *J*=6 Hz, *meso*‐Ar), 8.60 (d, 2 H, *J*=3 Hz, β‐pyrr), 8.74 (d, 2 H, *J*=3 Hz, β‐pyrr), 9.03 (br d, 4 H, β‐pyrr); Anal. Calcd for C_45_H_38_N_4_O_6_: C, 73.96; H, 5.24; N, 7.67; found: C, 73.91; H, 5.29; N, 7.62; MS (FAB, *m*/*z*): 733 (M^+^); UV‐vis (CH_2_Cl_2_): *λ*_max_ nm (log *ϵ*):425 (4.71), 580 (3.88), 621 (3.86), 651 (3.83).

**General procedure for the syntheses of Corr‐Pc dyads**: Corrole (0.033 mmol), phthalocyanine **4 a** (70 mg, 0.040 mmol), Cs_2_CO_3_ (12 mg, 0.035 mmol), Pd(OAc)_2_ (2 mg, 8.3×10^−3^ mmol), and PPh_3_ (2 mg, 8.3×10^−3^ mmol) were placed under argon atmosphere in a dry Schlenk flask. Dry DMSO was then added and three freeze‐pump‐thaw cycles were performed. The reaction mixture was stirred at 80 °C for 24 h. When no more corrole was detected by TLC analysis, the solvent was removed under reduced pressure and the residue was chromatographed (Silica gel, THF/Hexane 2:1), then a further purification by size‐exclusion chromatography on BioBeads SX1 (BioRad) using CH_2_Cl_2_ as eluent was performed. Data of the newly prepared compounds are given below.

**Dyad 5 a**: Yield: 14 %, dark green solid, mp>200 °C. ^1^H NMR (400 MHz, [D_8_]THF, *δ* ppm): 0.88–1.12 (br m, 90 H, CH, CH_2_, CH_3_), 2.56 (br m, 12 H, SO_2_CH_2_), 4.08 (s, 6 H, OCH_3_),7.43 (br d, 4 H, *meso*‐Ar), 7.91 (d, 2 H, *J*=8 Hz, *meso*‐Ar), 8.16 (d, 2 H, *J*=8 Hz, *meso*‐Ar), 8.36 (d, 4 H, *J*=8 Hz, *meso*‐Ar), 8.61 (br m, 4 H, β‐pyrr), 8.91 (d, 2 H, *J*=4 Hz, β‐pyrr), 9.00 (br d, 2 H, β‐pyrr), 9.98 (s, 1 H, Pc‐H), 10.07 (br m, 2 H, Pc‐H), 10.23 (br m, 6 H, Pc‐H); Anal. Anal. Calcd for C_121_H_140_N_12_O_14_S_6_Zn: C, 64.76; H, 6.29; N, 7.49; found: C, 64.84; H, 6.31; N, 7.50; MS (MALDI‐TOF, matrix dithranol *m*/*z*): 2242.8 (M^+^); UV‐vis (CH_2_Cl_2_): *λ*_max_ (nm) (log *ϵ*)=393 (4.73), 435 (4.70), 628 (sh), 652 (sh), 687 (4.78), 707 (4.77).

**Dyad 5 b**: Yield: 22 %. Dark blue solid. mp>200 °C. ^1^H NMR (400 MHz, [D_8_]THF, *δ* ppm): 0.85–1.11 (br m, 90 H, CH, CH_2_, CH_3_), 2.54 (br m, 12 H, SO_2_CH_2_), 3.89 (s, 12 H, OCH_3_), 3.96 (s, 6 H, OCH_3_), 7.56 (br m, 4 H, *meso*‐Ar), 7.94 (br d, 2 H, *meso*‐Ar), 8.19 (d, 2 H, *J*=8 Hz, *meso*‐Ar), 8.55 (br d, 4 H, β‐pyrr), 8.91 (br d, 4 H, β‐pyrr); 9.24 (s, 1 H, Pc‐H), 10.47 (br m, 2 H, Pc‐H), 10.64 (br m, 6 H, Pc‐H); Anal. Calcd for C_125_H_148_N_12_O_18_S_6_Zn: C, 63.50; H, 6.31; N, 7.11; found: C, 63.45; H, 6.33; N, 7.20; MS (MALDI‐TOF, matrix dithranol *m*/*z*): 2363.8 (M^+^); UV‐vis (CH_2_Cl_2_): *λ*_max_ (nm) (log *ϵ*)=429 (4.56), 628 (sh), 650 (sh), 684 (4.50), 709 (4.53).

## Conflict of interest

The authors declare no conflict of interest.

## Supporting information

As a service to our authors and readers, this journal provides supporting information supplied by the authors. Such materials are peer reviewed and may be re‐organized for online delivery, but are not copy‐edited or typeset. Technical support issues arising from supporting information (other than missing files) should be addressed to the authors.

Supplementary
