# Supplementary material for: Panchromatic Light Harvesting and Stabilizing Charge‐Separated States in Corrole–Phthalocyanine Conjugates through Coordinating a Subphthalocyanine
Source: Chemistry. 2020 Sep 21;26(59):13451–61. doi: 10.1002/chem.202001442 (PMC7693288; doi:10.1002/chem.202001442)
Supplement: Supplementary file 1 — Supplementary [file CHEM-26-13451-s001.pdf]

Chemistry–A European Journal

Supporting Information

Panchromatic Light Harvesting and Stabilizing Charge-Separated States in Corrole–Phthalocyanine Conjugates through Coordinating a Subphthalocyanine

Beatrice Berionni Berna,[a, b, c] Benedikt Platzer,[e] Maximiliam Wolf,[e] Giulia Lavarda,[b, c] Sara Nardis,[a] Pierluca Galloni,[a] Tomás Torres,*[b, c, d] Dirk M. Guldi,*[e] and Roberto Paolesse*[a]

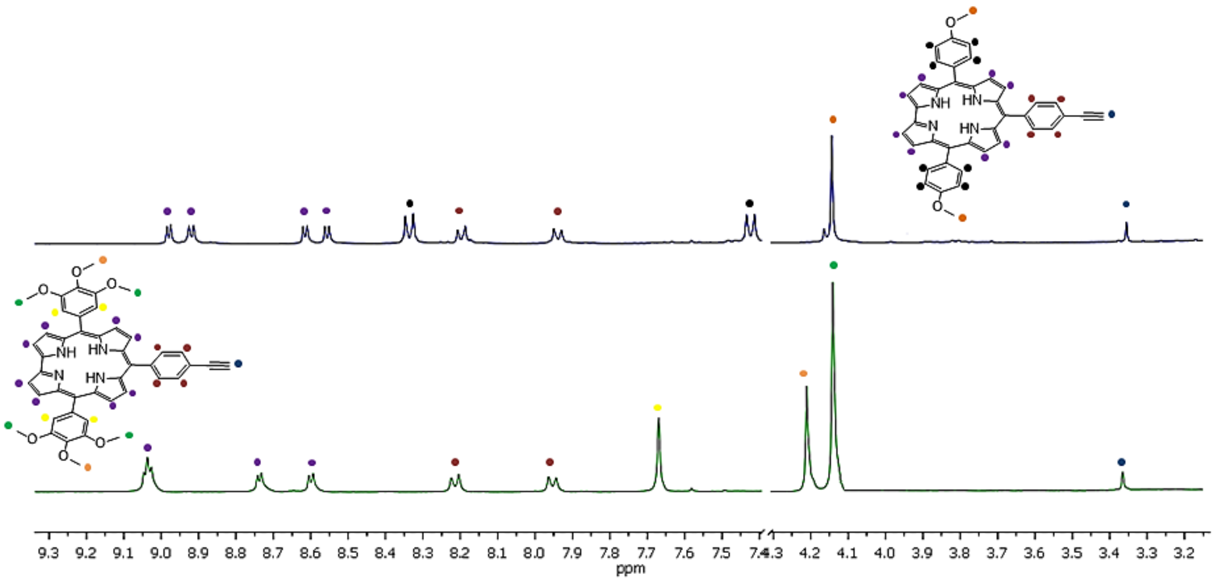

Figure S1. ^{1}H NMR spectra of **3a** and **3b**

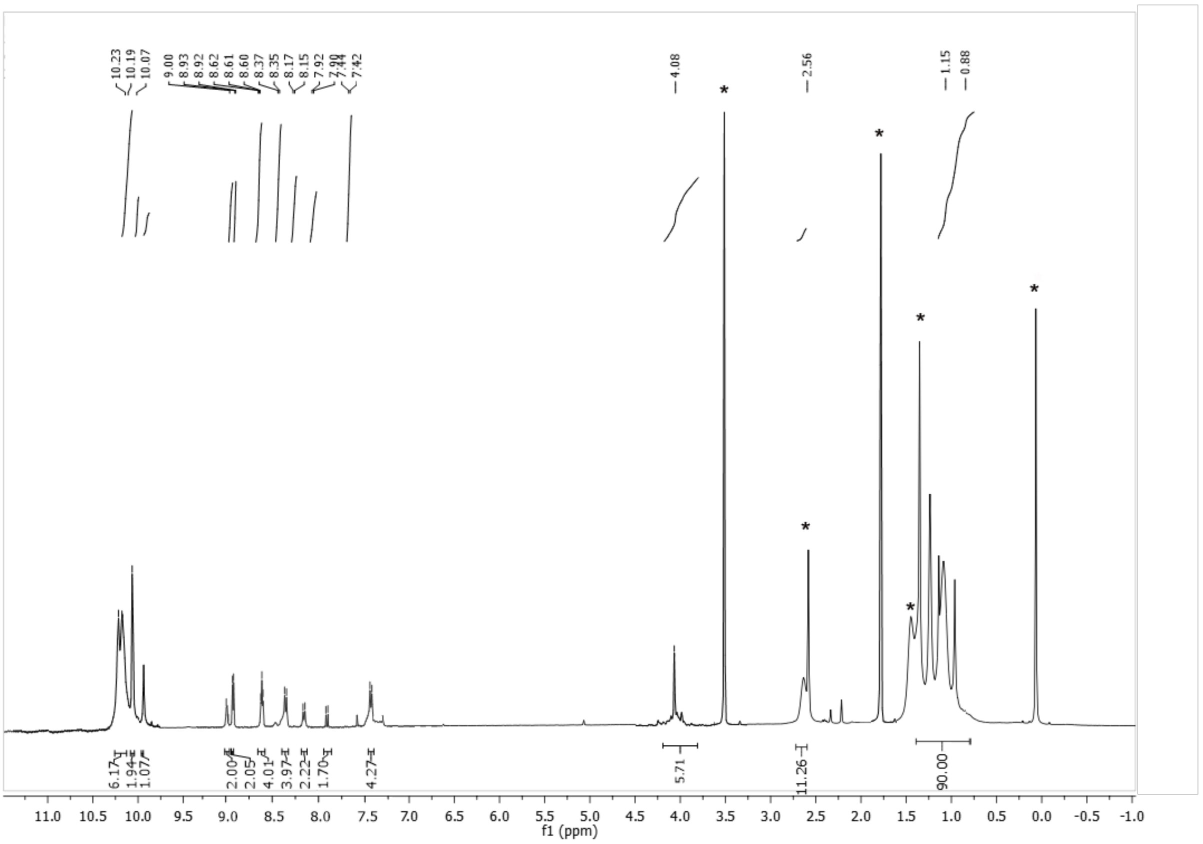

Figure S2. ^{1}H NMR spectra of **5a** (asterisks indicate residual solvent peaks)

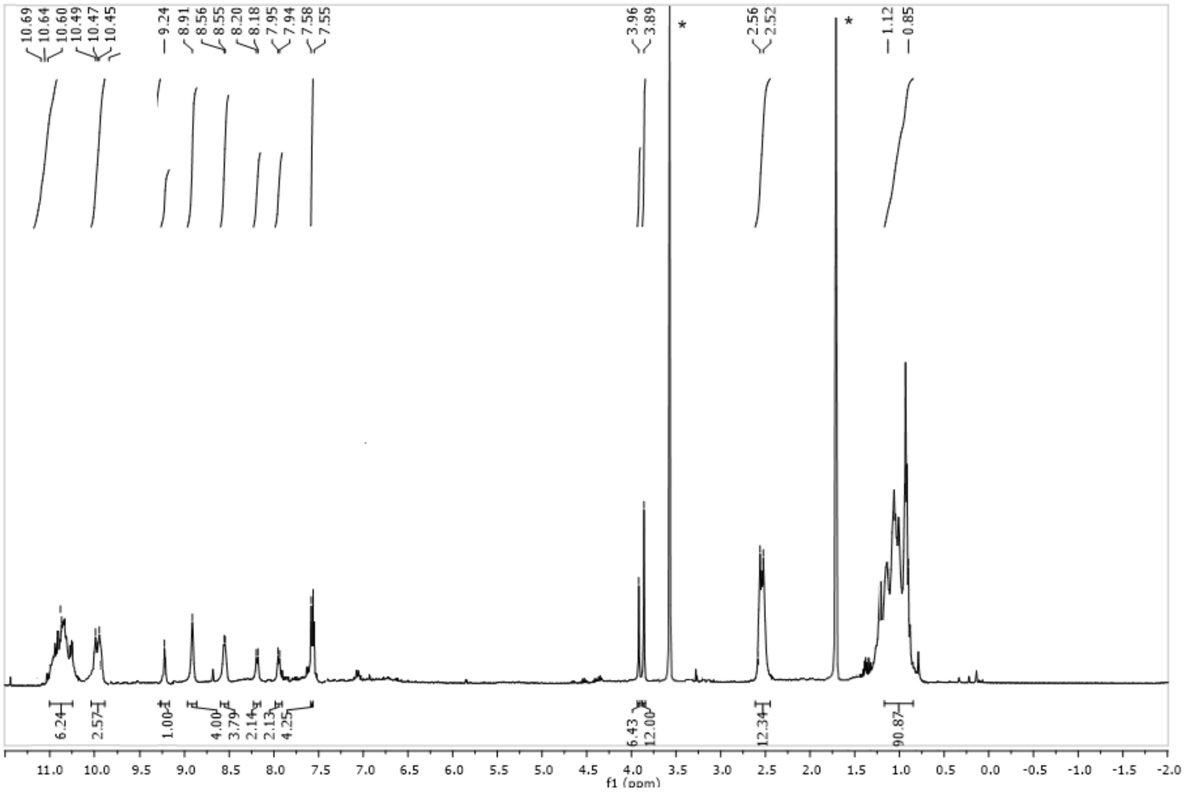

Figure S3. ^{1}H NMR spectra of **5b** (asterisks indicate residual solvent peaks)

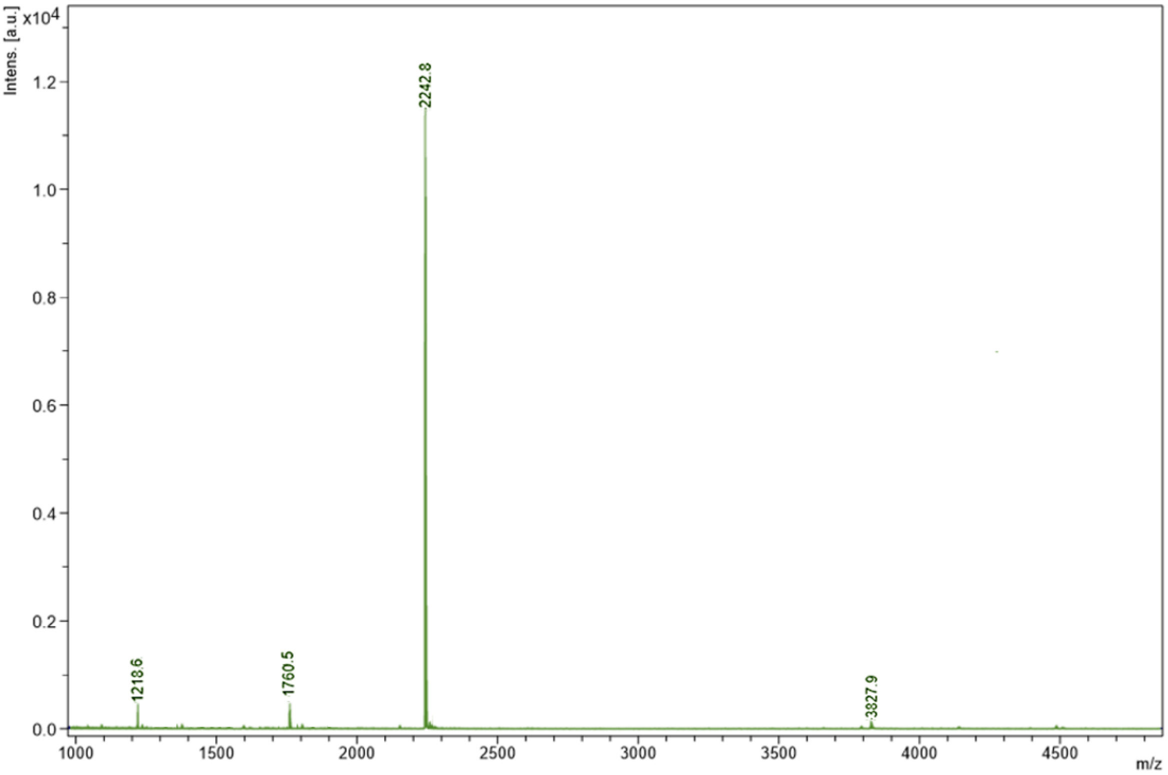

Figure S4. MALDI-TOF spectrum of dyad **5a.**

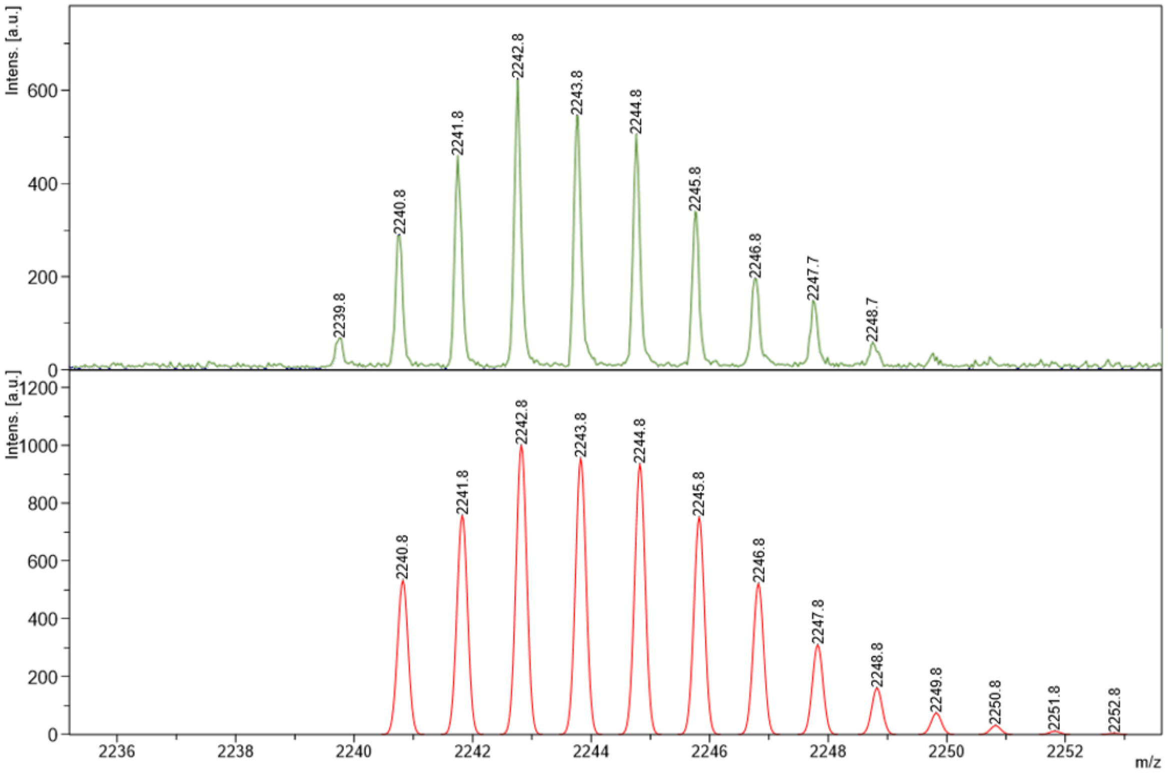

Figure S5. High-resolution MALDI-TOF mass spectrum of **5a**. Theoretical (bottom) and experimental (top) pattern found for the ion molecular peak $[M]^+$.

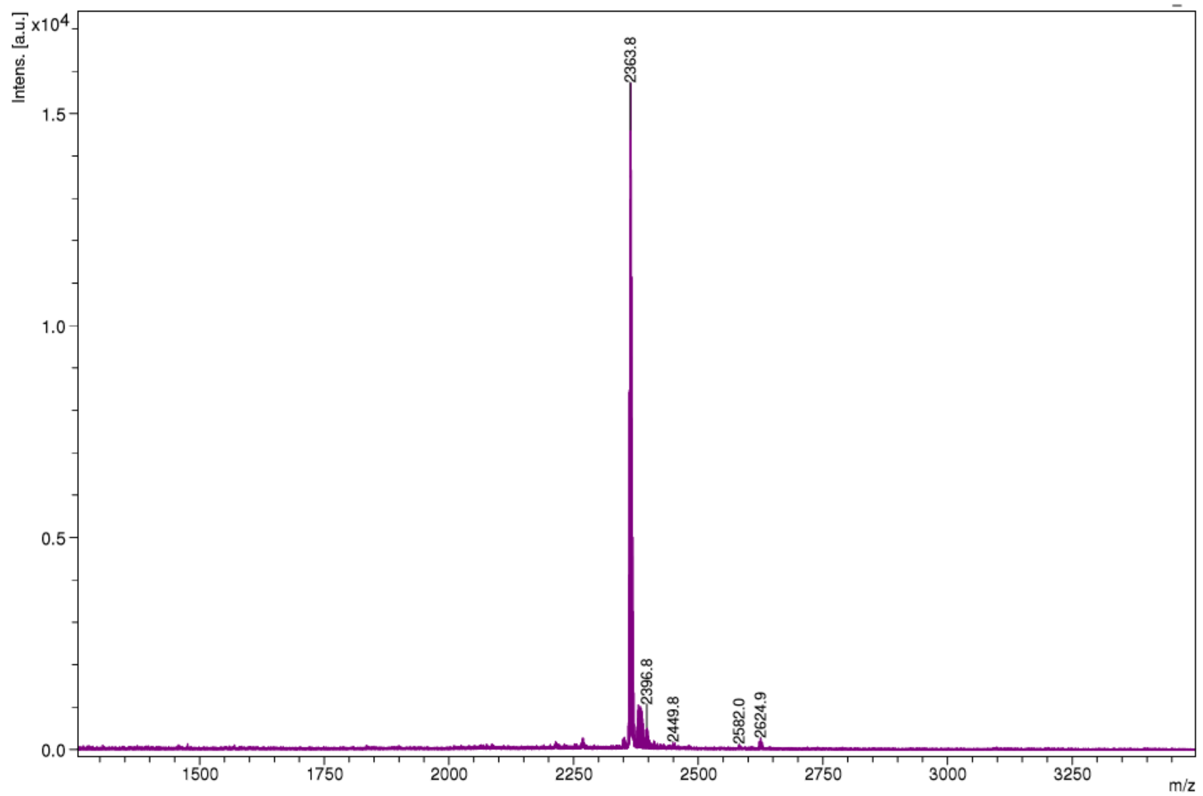

Figure S6. MALDI-TOF spectrum of dyad **5b.**

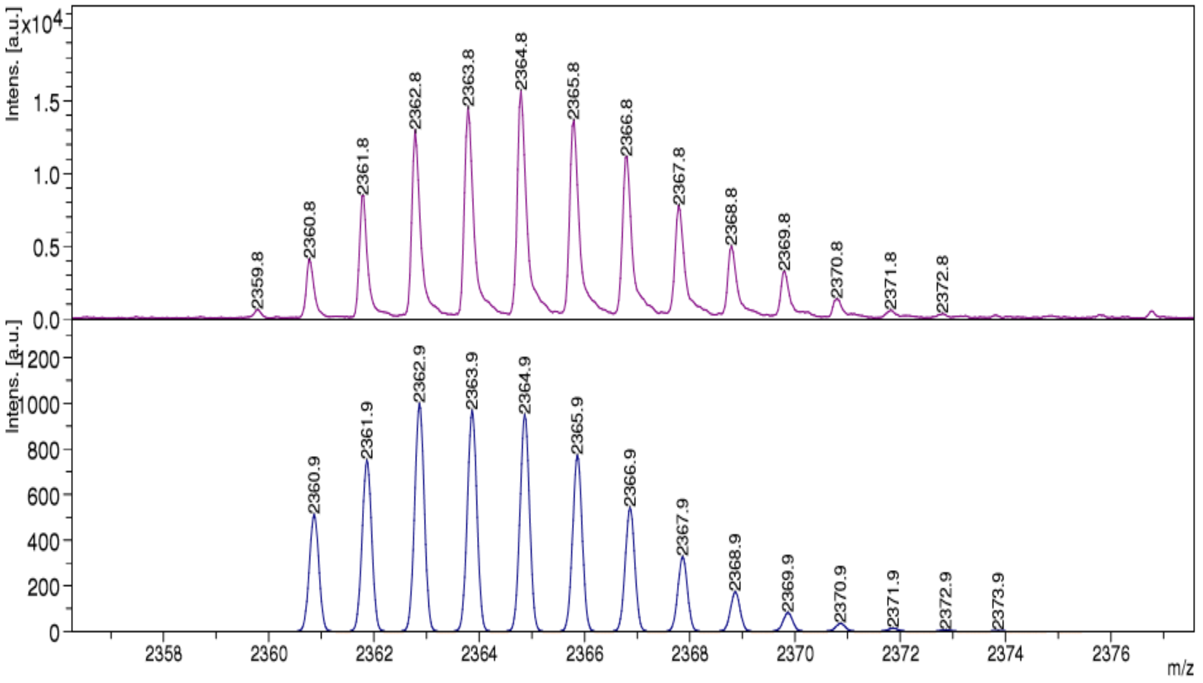

Figure S7. High-resolution MALDI-TOF mass spectrum of **5b**. Theoretical (bottom) and experimental (top) pattern found for the ion molecular peak $[M]^+$.

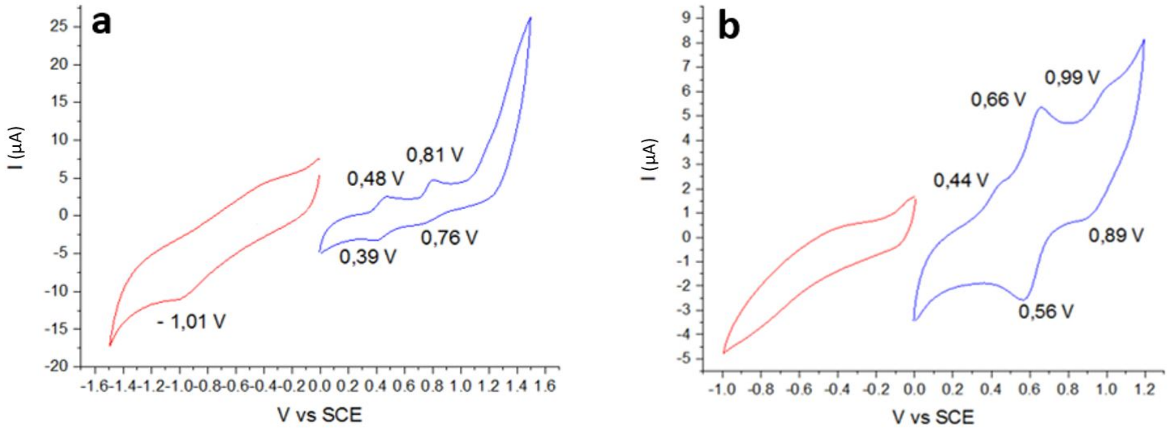

Figure S8. Cyclic voltammograms of (a) **3a** and (b) **3b** in DCM including 0.1 M TBAP as electrolyte acquired at room-temperature (Scan-Rate: 0.1 V/s). Voltage is given vs. a standard calomel electrode.

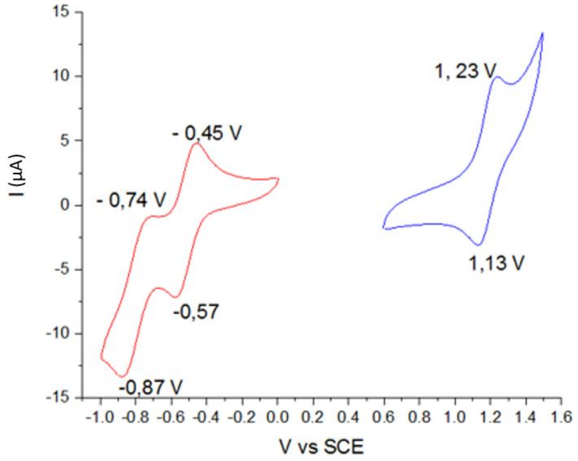

Figure S9. Cyclic voltammograms of **4b** in DCM including 0.1 M TBAP as electrolyte acquired at room-temperature (Scan-Rate: 0.1 V/s). Voltage is given vs. a standard calomel electrode.

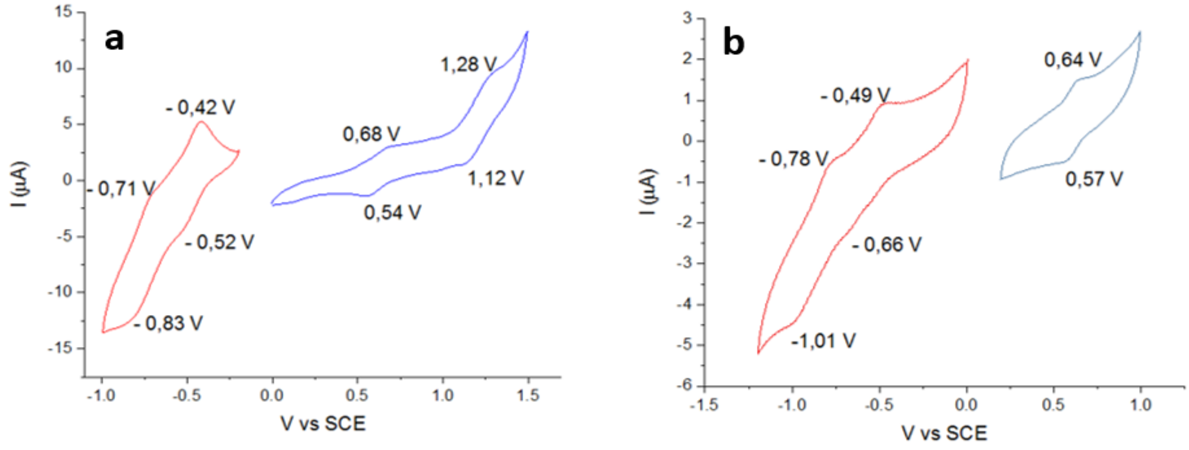

Figure S10. Cyclic voltammograms of (a) **5a** and (b) **5b** in DCM including 0.1 M TBAP as electrolyte acquired at room-temperature (Scan-Rate: 0.1 V/s). Voltage is given vs. a standard calomel electrode.

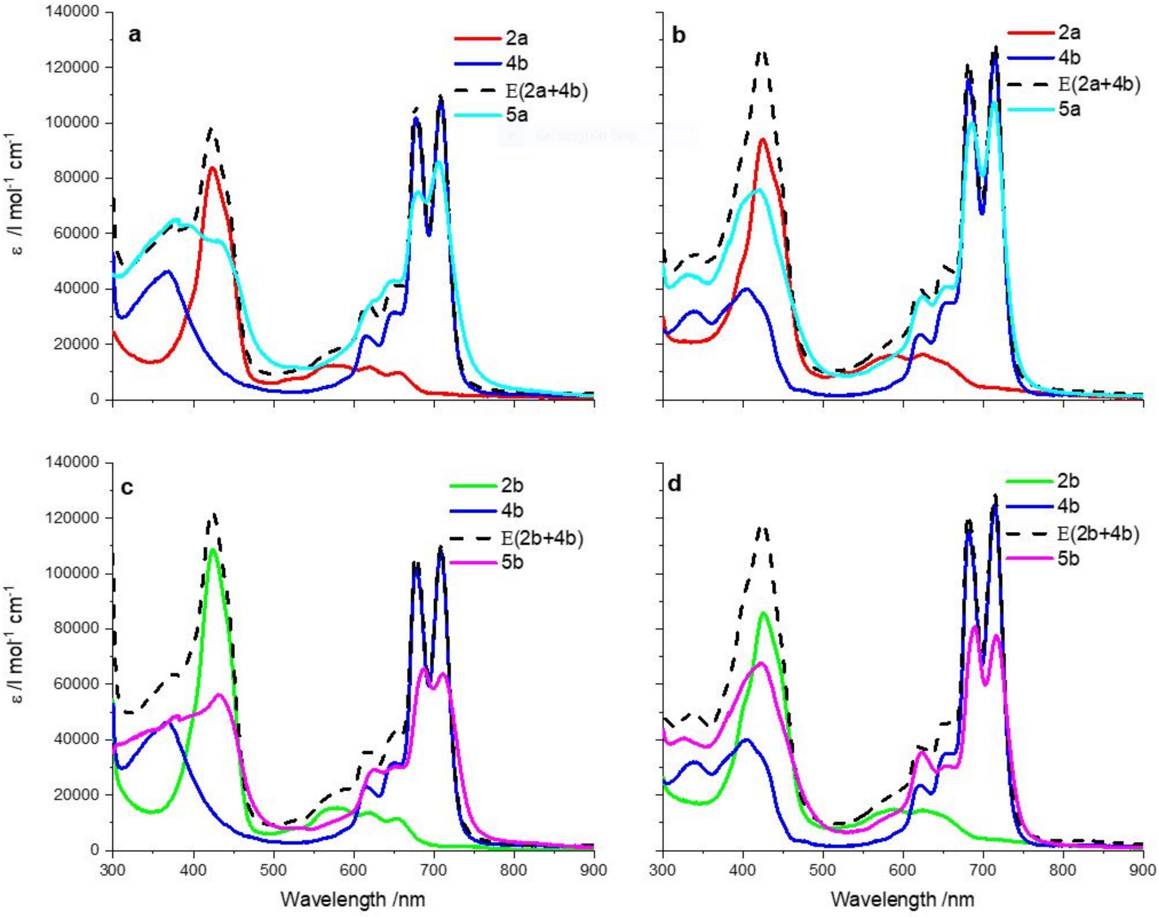

Figure S11. Steady-state absorption spectra of the dyads **5a** (a, b) and **5b** (c, d), corresponding references and combined references in anisole (left column: a, c) and PhCN (right column: b, d) at room-temperature.

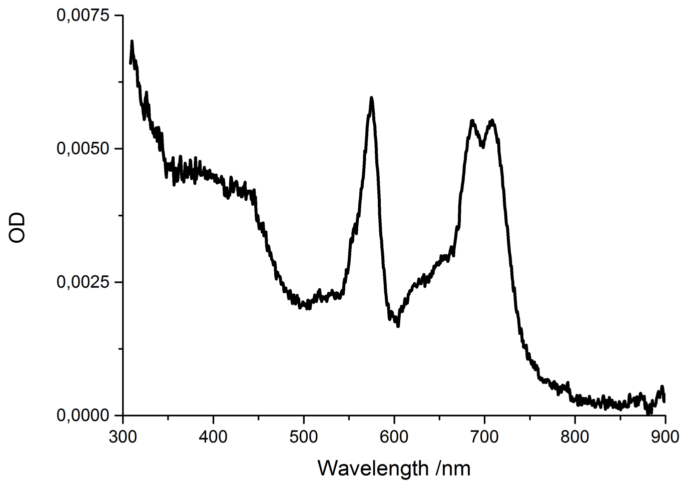

Figure S12. Steady-state absorption spectrum of a 1:1 **5a**/**6** mixture in anisole at room-temperature.

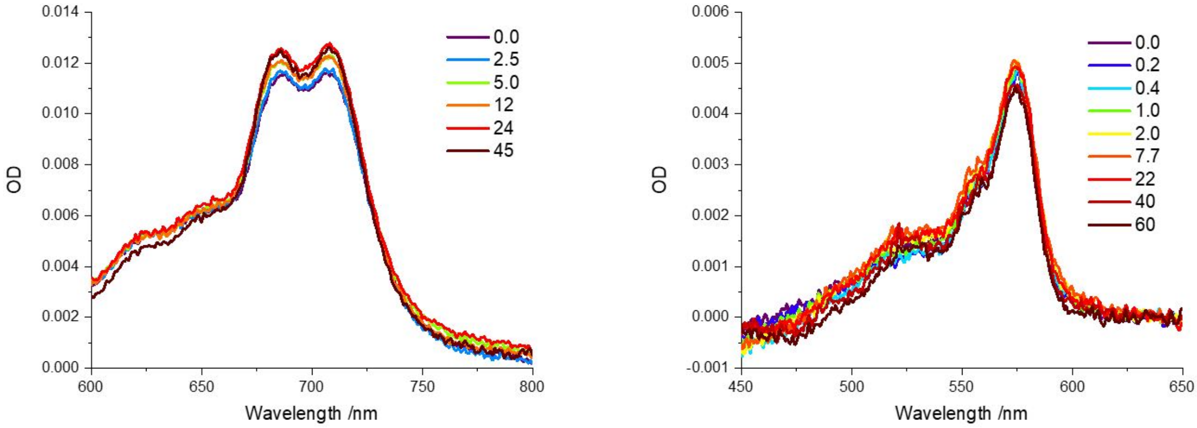

Figure S13. Steady-state absorption spectra of **5a** upon addition of **6** with given **6**:**5a**-ratios (left) and **6** upon addition of **5a** with given **5a**:**6**-ratios (right) in anisole at room-temperature. All spectra were corrected for the respective additive substances, **6** and **5a**.

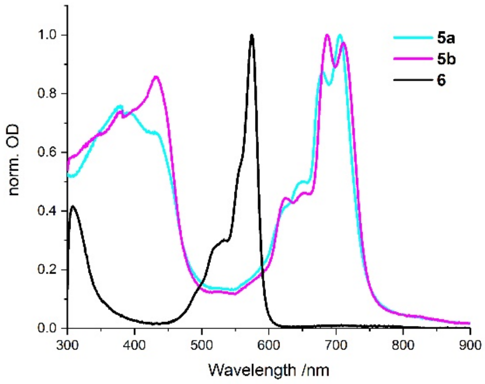

Figure S14. Normalized steady-state absorption spectra of the dyads **5a,b** and SubPc **6** in anisole at room-temperature. In combination the resulting supramolecular triads feature panchromatic absorption.

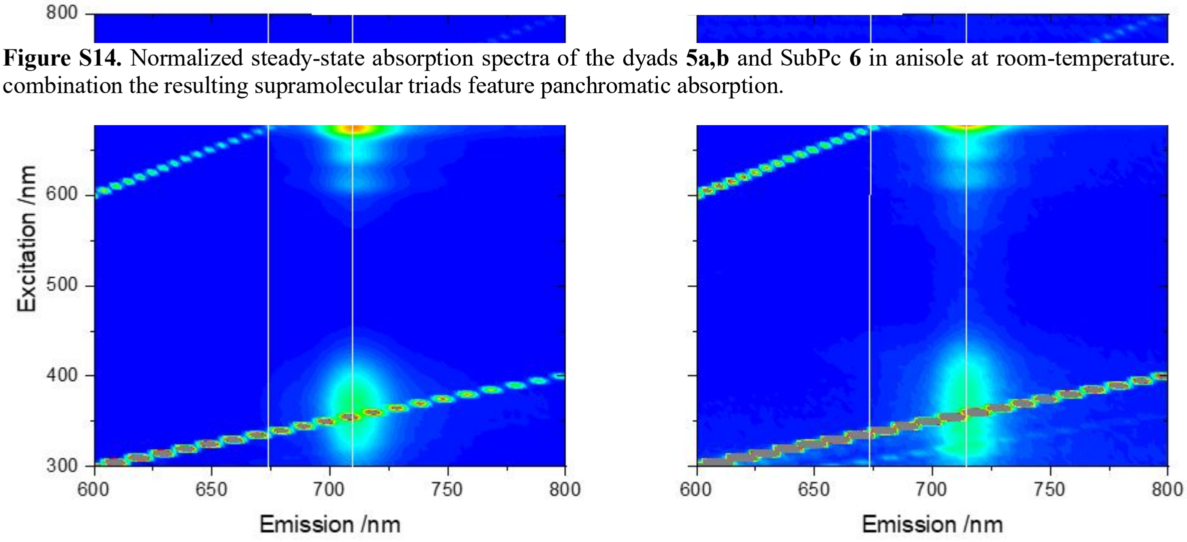

Figure S15. 3D Steady-state fluorescence plots with excitation from 300 nm to 800 nm and detected emission from 600 nm to 800 nm for dyads (left) **5a** and (right) **5b** in toluene at room temperature. The white vertical lines signal (from left to right) the position of expected maxima for the corrole (674 nm) and phthalocyanine moiety (611/614 nm), respectively.

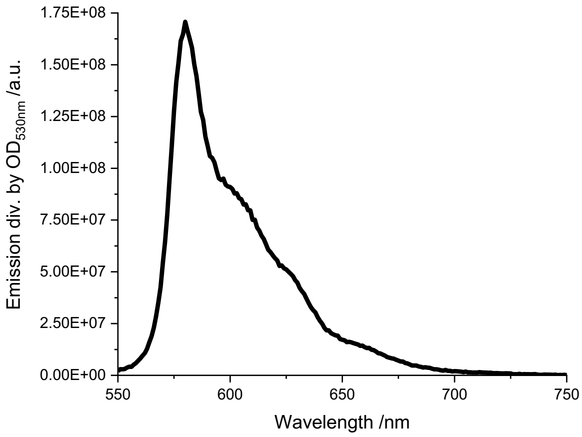

Figure S16. Steady-state fluorescence spectrum of SubPc **6** upon excitation at 530 nm with given emission intensity divided by the respective OD at the excitation in anisole and acquired at room-temperature.

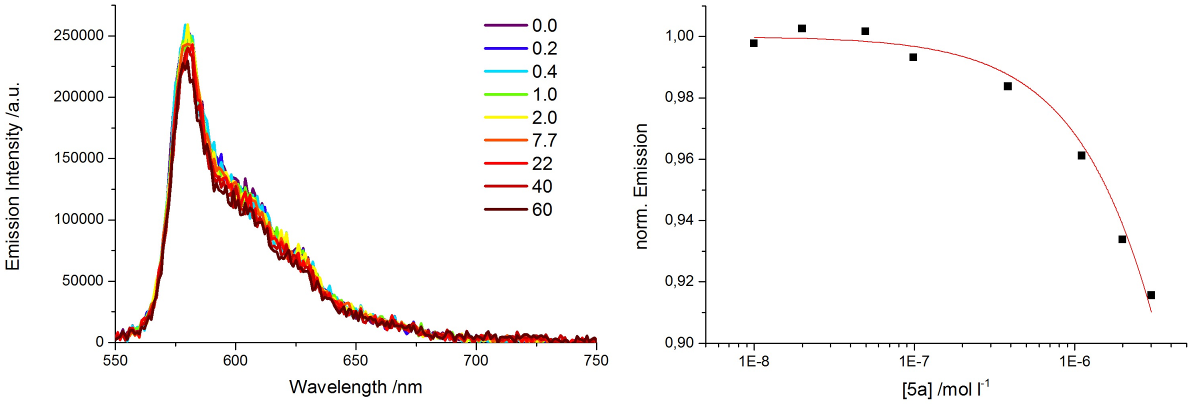

Figure S17. Steady-state fluorescence spectra of SubPc **6** upon addition of **5a** with given **5a**:**6**-ratios in anisole at room-temperature (left) and corresponding total emission vs. concentration of the additive substance **5a** (right).

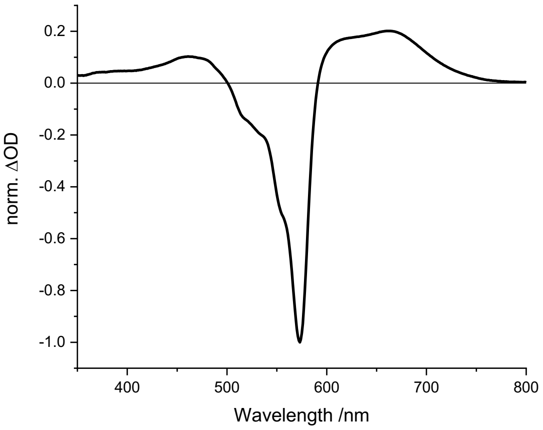

Figure S18. Differential absorption spectra obtained upon electrochemical reduction of SubPc **6** at an applied bias of -0.8 V in ODCB (including 0.1 M n-Bu_4NClO_4 as electrolyte) after solvent-saturation with Argon at room-temperature and silver-wire as pseudo-reference electrode.

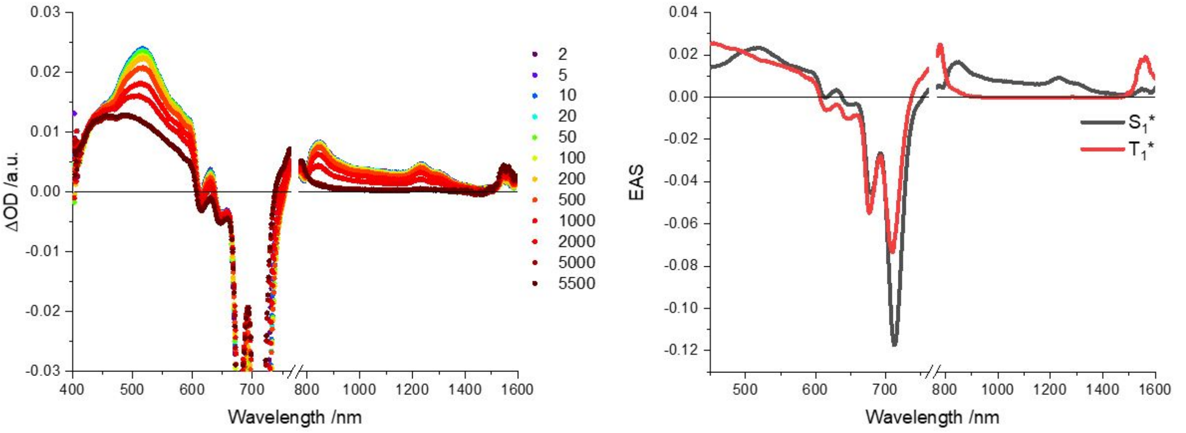

Figure S19. (left) Differential absorption spectra with delays between 2 and 5500 ps and (right) evolution associated spectra of transient species of **4b** obtained upon femtosecond flash-photolysis (excitation at 676 nm) in de-aerated anisole at room-temperature.

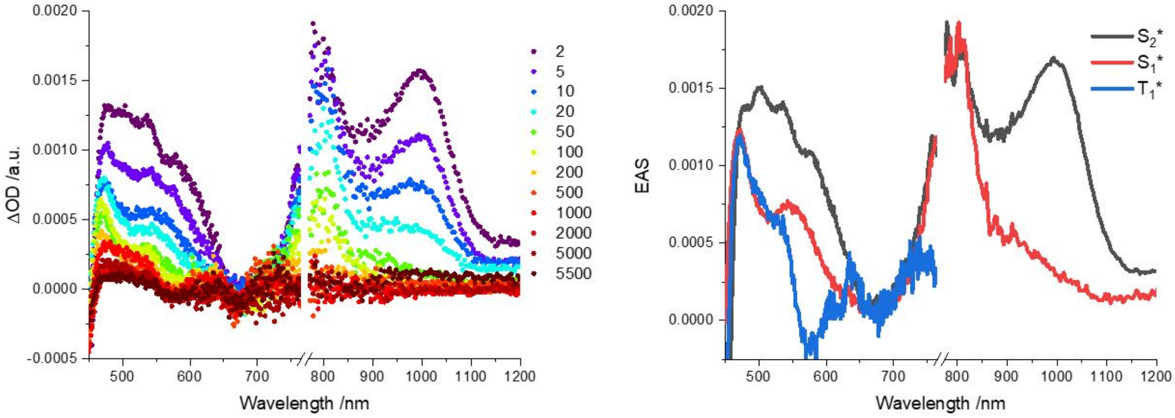

Figure S20. (left) Differential absorption spectra with delays between 2 and 5500 ps and (right) evolution associated spectra of transient species of **2a** obtained upon femtosecond flash-photolysis (excitation at 430 nm) in de-aerated anisole at room-temperature.

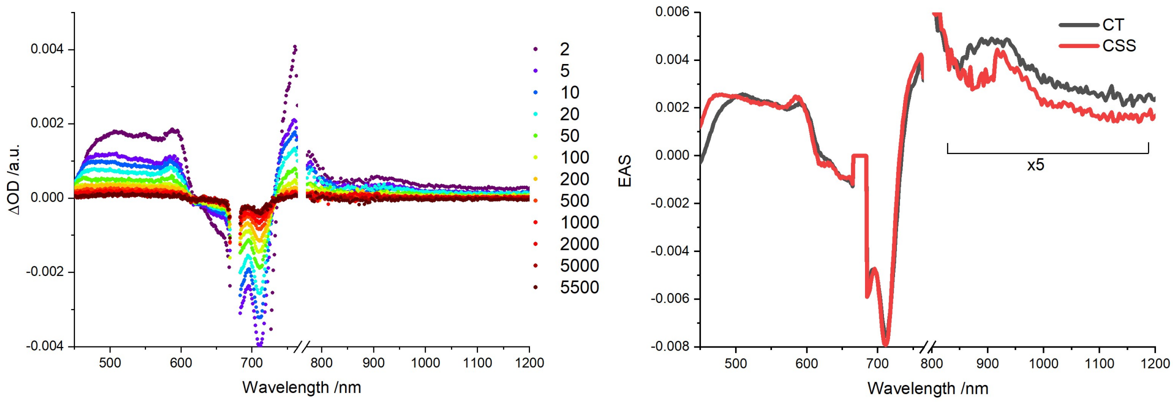

Figure S21. (left) Differential absorption spectra with delays between 2 and 5500 ps and (right) evolution associated spectra of transient species of **5a** obtained upon femtosecond flash-photolysis (excitation at 676 nm) in de-aerated anisole at room-temperature.

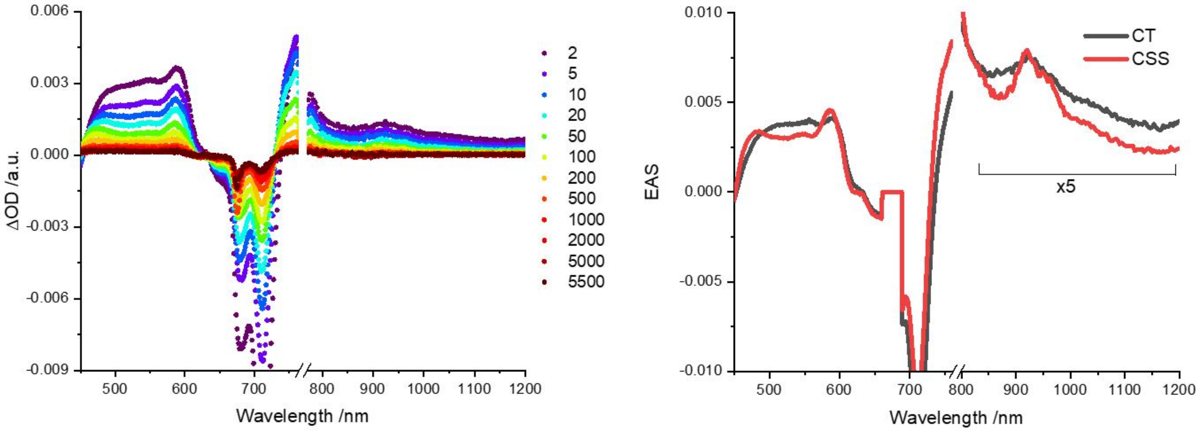

Figure S22. (left) Differential absorption spectra with delays between 2 and 5500 ps and (right) evolution associated spectra of transient species of **5b** obtained upon femtosecond flash-photolysis (excitation at 676 nm) in de-aerated anisole at room-temperature.

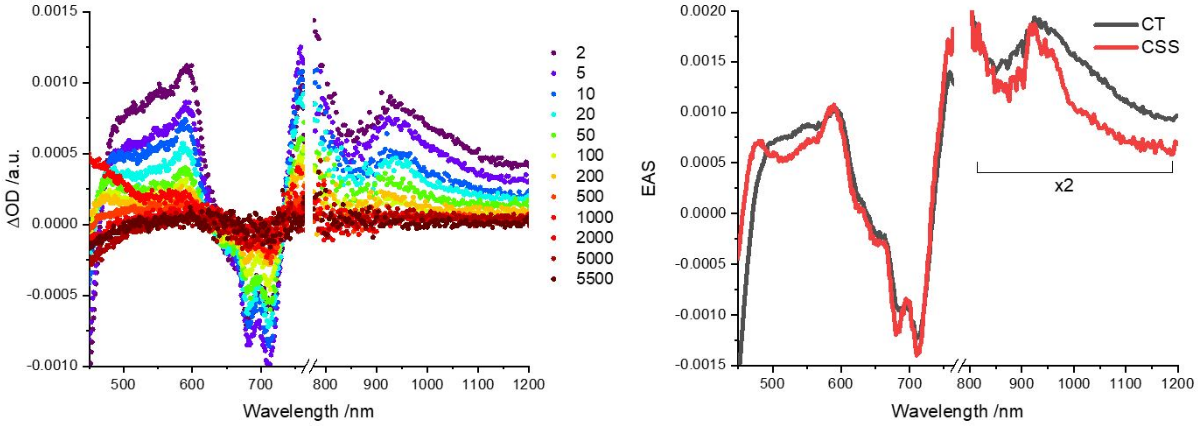

Figure S23. (left) Differential absorption spectra with delays between 2 and 5500 ps and (right) evolution associated spectra of transient species of **5b** obtained upon femtosecond flash-photolysis (excitation at 430 nm) in de-aerated anisole at room-temperature.

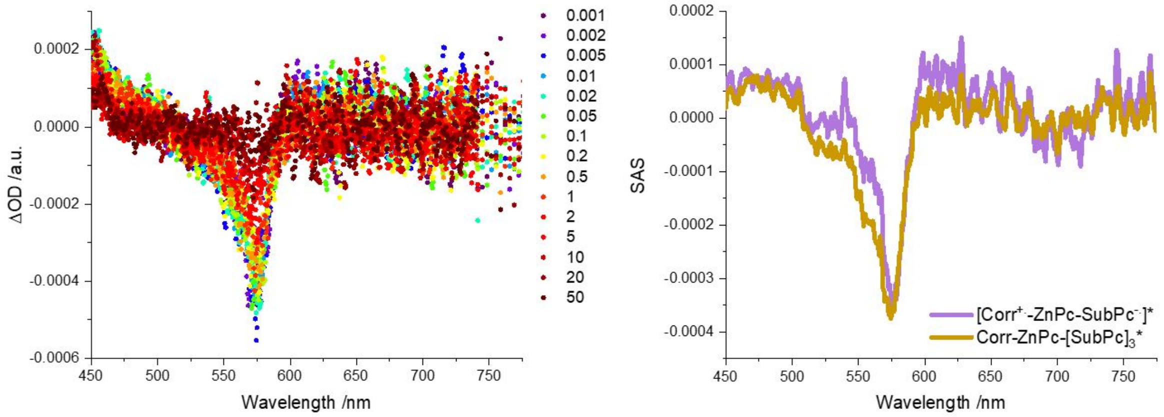

Figure S24. (left) Differential absorption spectra with delays between 0.001 and 50 µs and (right) species associated spectra of transient species of a 1:20 mixture of **5a** and **6** obtained upon nanosecond flash-photolysis (excitation at 430 nm) in de-aerated anisole at room-temperature.

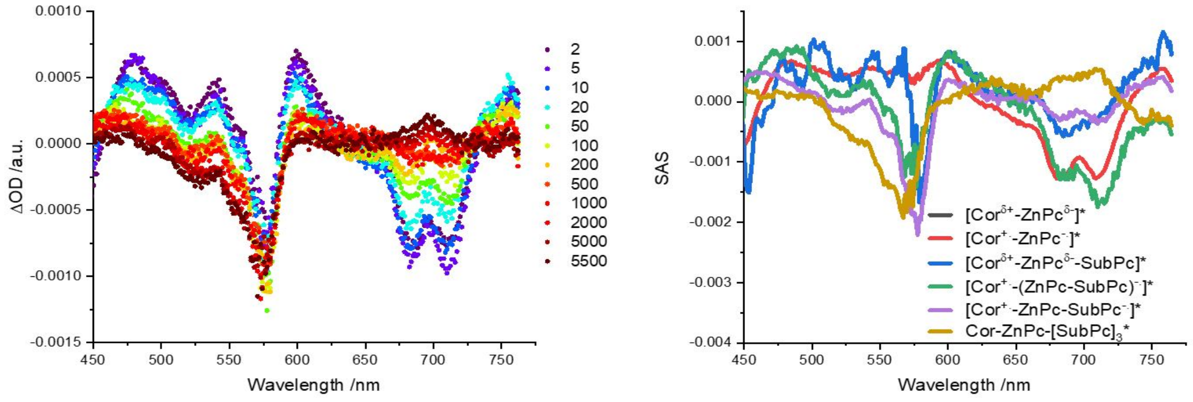

Figure S25. (left) Differential absorption spectra with delays between 2 and 5500 ps and (right) species associated spectra of transient species of a 1:20 mixture of **5b** and **6** obtained upon femtosecond flash-photolysis (excitation at 430 nm) in de-aerated anisole at room-temperature.

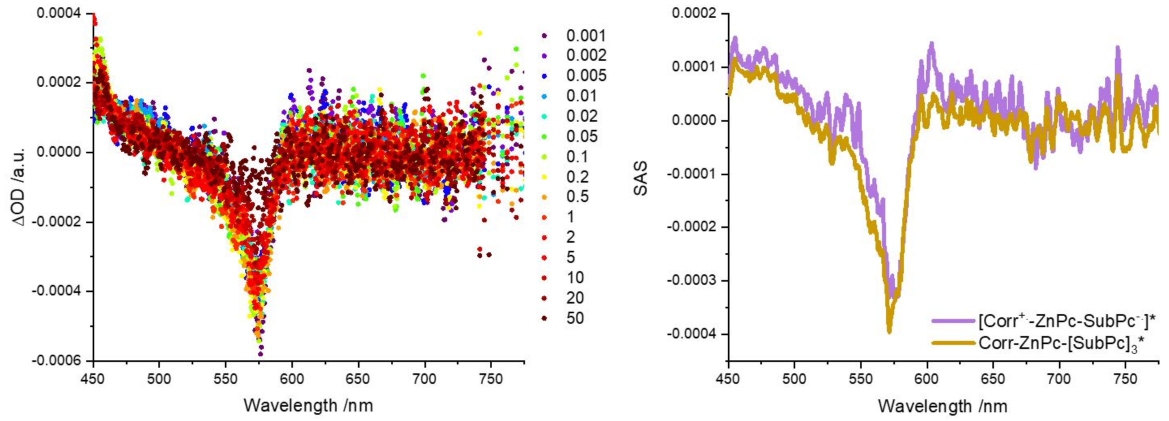

Figure S26. (left) Differential absorption spectra with delays between 0.001 and 50 μs and (right) species associated spectra of transient species of a 1:20 mixture of **5b** and **6** obtained upon nanosecond flash-photolysis (excitation at 430 nm) in de-aerated anisole at room-temperature.

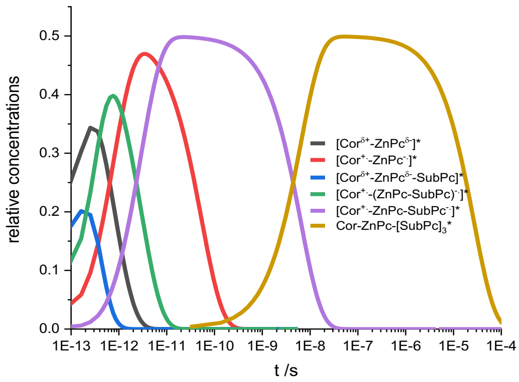

Figure S27. Relative concentration profiles of observed transient species obtained from femtosecond and nanosecond flash-photolysis (excitation at 430 nm) of a 1:20 mixture of **5b** and **6** in de-aerated anisole at room-temperature.

Table S1. Summarized excited state lifetimes gained upon deconvolution of femtosecond and nanosecond flash-photolysis data by means of global and target analysis. Measurements were performed in de-aerated anisole at room-temperature.

Sample	Excitation /nm	τ_1	τ_2	τ_3	τ_4
**2a**	430	4 ps (*S2)	80 ps (*S1)	> 5 ns (*T1)	
**4b**	676	2.7 ns (*S1)	≥ ns (*T1)		
**5a**	430	1 ps (*CT)	55 ps (*CSS)		
	676	1 ps (*CT)	70 ps (*CSS)		
**5b**	430	7 ps (*CT)	120 ps (*CSS)		
	676	5 ps (*CT)	90 ps (*CSS)		
**5a/6**	430	~ 0.2 ps (*CT)	6 ps (short *CSS)	4.0 ns (long *CSS)	~ µs (*T1)
**5b/6**	430	~ 0.2 ps (*CT)	3 ps (short *CSS)	6.4 ns (long *CSS)	~ µs (*T1)
